# Supplementary material for: The glycosylation design space for recombinant lysosomal replacement enzymes produced in CHO cells
Source: Nat Commun. 2019 Apr 30;10:1785. doi: 10.1038/s41467-019-09809-3 (PMC6491494; doi:10.1038/s41467-019-09809-3)
Supplement: Supplementary file 1 — Supplementary Information [file 41467_2019_9809_MOESM1_ESM.pdf]

The Glycosylation Design Space for Recombinant Lysosomal Replacement Enzymes Produced in CHO Cells

Tian *et al.*

Supplementary Information

Supplementary Figure 1. RNAseq transcriptome profiling of CHO cells showing predicted expression of selected genes.

Supplementary Figure 2. Detailed presentation of N-glycans identified by site-specific N-glycan profiling of GLA produced in CHO WT and engineered KO/KI clones as indicated.

Supplementary Figure 3. Characterization of growth and yield performance of glycoengineered CHO clones expressing GLA.

Supplementary Figure 4. Detailed presentation of N-glycans identified by site-specific N-glycan profiling of GBA produced in CHO WT and engineered KO clones as indicated.

Supplementary Figure 5. Targeted MS/MS manual annotation of the most representative glycopeptides.

Supplementary Figure 6. In vitro assay of enzyme specific activity and plasma stability of GLA glycovariants.

Supplementary Figure 7. SDS-PAGE Western blot analysis of infused GLA glycovariants in mice plasma.

Supplementary Figure 8. Relative distribution of GLA glycovariants among the four major visceral organs.

Supplementary Table 1. CRISPR gRNA design and list of PCR primers used for gRNA target sites.

Supplementary Table 2. Summary of CHO mutant clones stably expressing GLA and cell line ancestry.

Supplementary Table 3. Summary of CHO mutant clones stably expressing GBA and cell line ancestry.

Supplementary Table 4. Sequence analysis of CHO mutant clones stably expressing GBA.

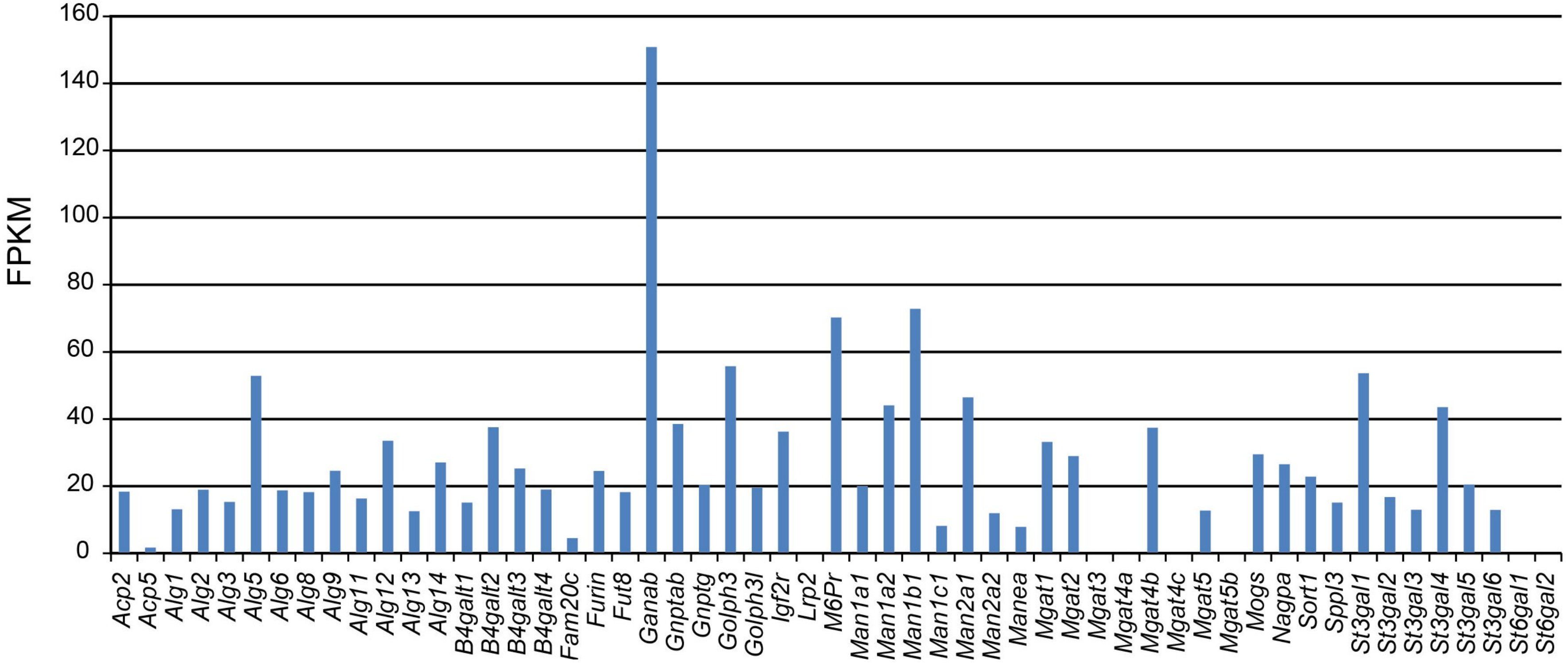

Supplementary Figure 1. RNAseq transcriptome profiling of CHO cells showing predicted expression of select genes studied.

RNAseq analysis was performed with CHO GS$^{-/-}$ cells as previously reported[1]. Genes known to function in N-glycosylation and M6P-tagging, including glycosyltransferases, glycosylhydrolases, enzymes involved in dolichol-linked precursor oligosaccharide synthesis, and other related genes, are shown. Source data are provided as a Source Data file.

[1]Yang, Z. et al. Engineered CHO cells for production of diverse, homogeneous glycoproteins. *Nat. Biotechnol.* **33**, 842-844 (2015).

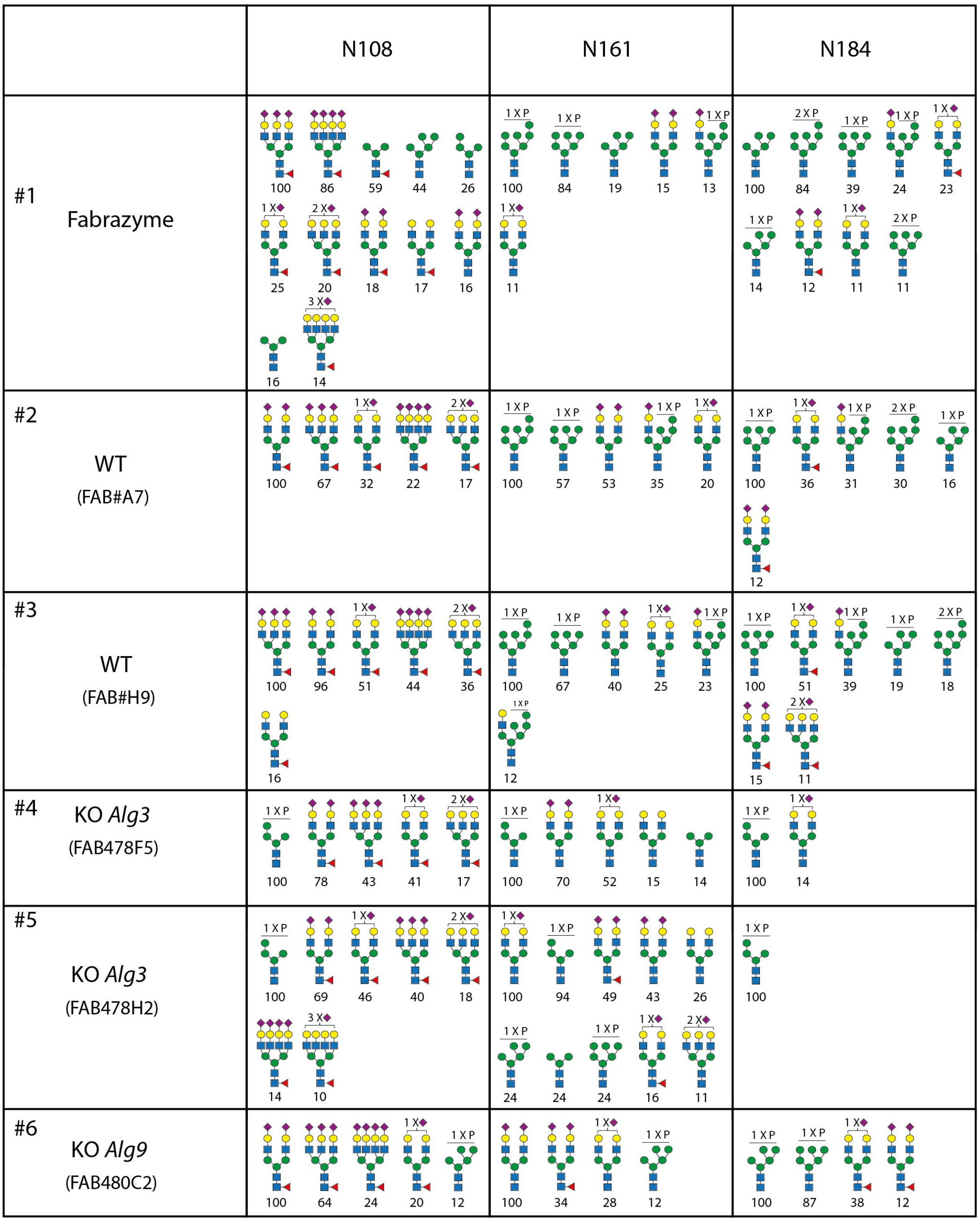

Supplementary Figure 2. Detailed presentation of N-glycans identified by site-specific N-glycan profiling of GLA produced in CHO WT and engineered KO/KI clones as indicated.

N-glycan structures and their relative abundances at each of the three N-glycosites (N108, N161, and N184) of GLA are illustrated with their relative abundance adjusted to the most abundant structure. Minor glycoforms identified with relative abundance less than 10% are not shown. Same N-glycan composition may represent isobaric structures.

(Page 1 of 12, continued)

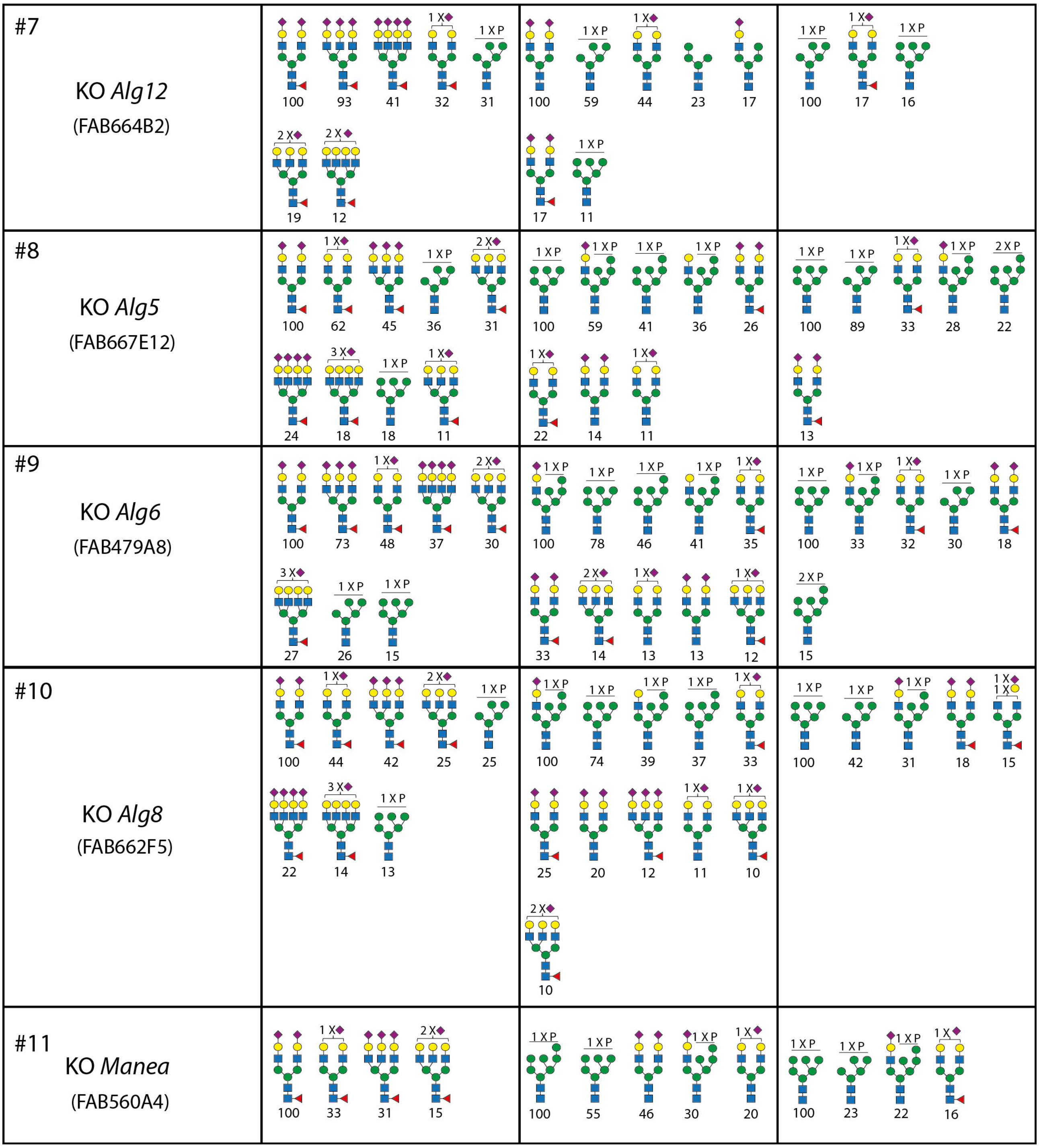

Supplementary Figure 2 continued.

(Page 2 of 12, continued)

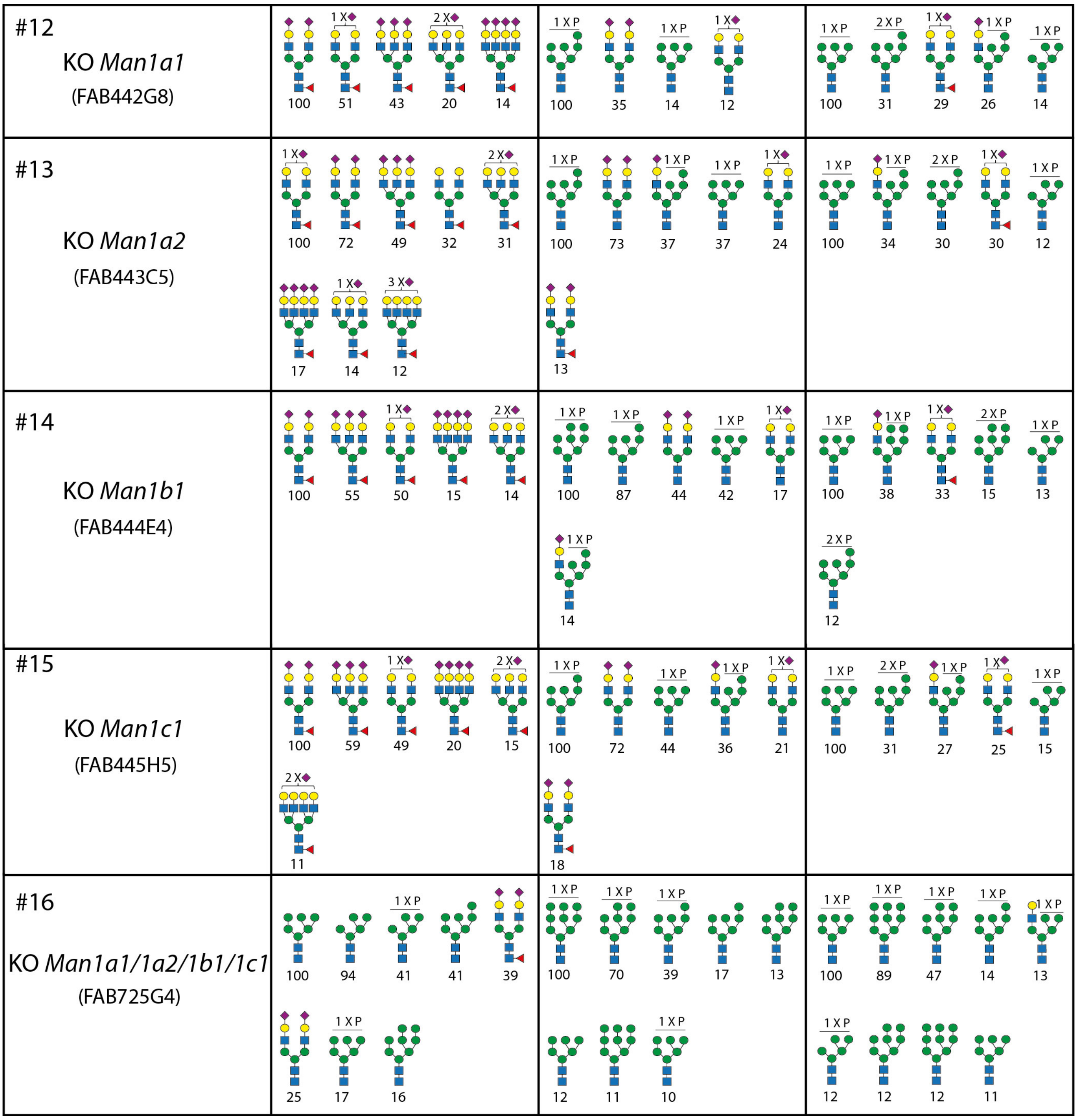

Supplementary Figure 2 continued.

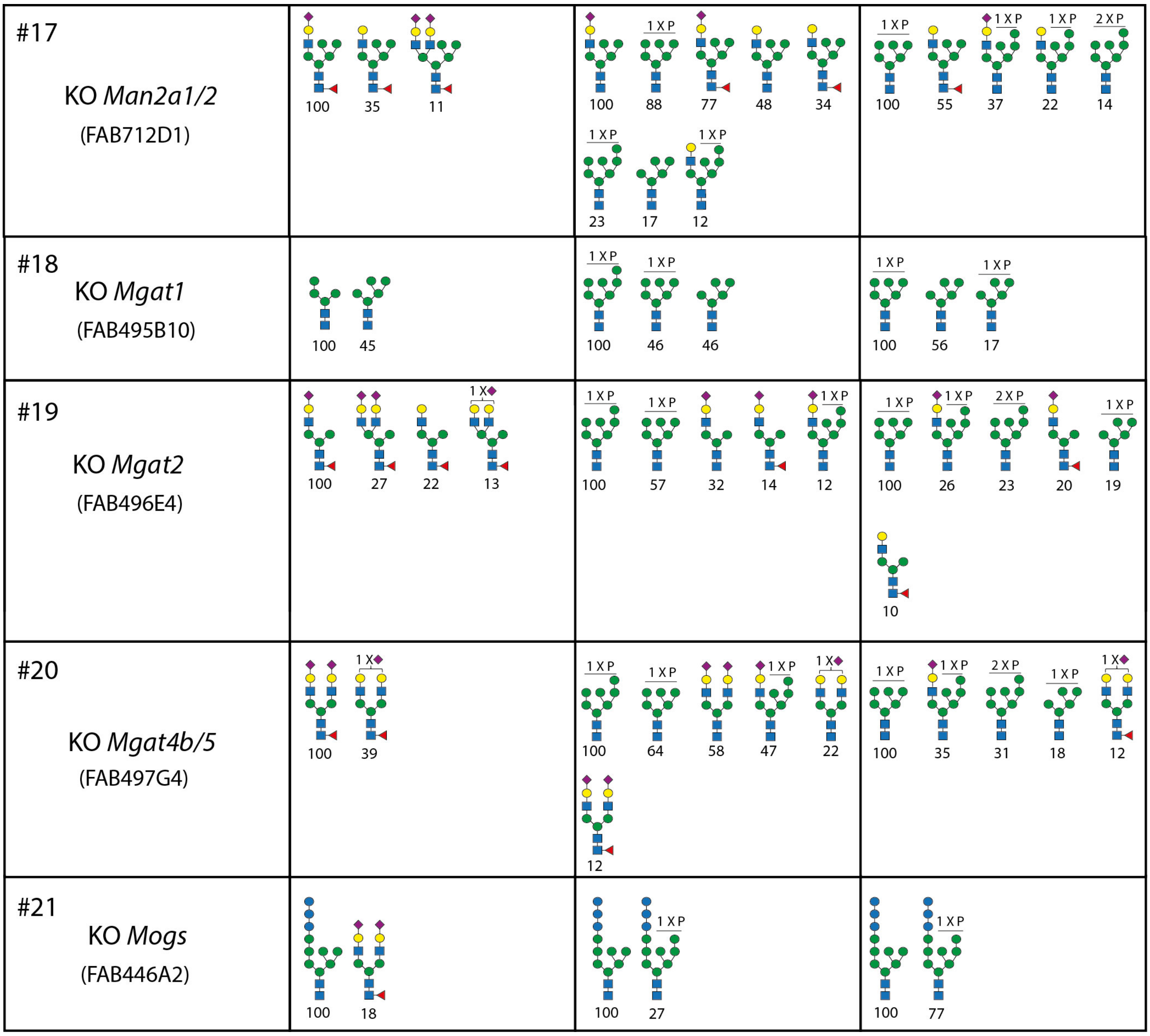

Supplementary Figure 2 continued.

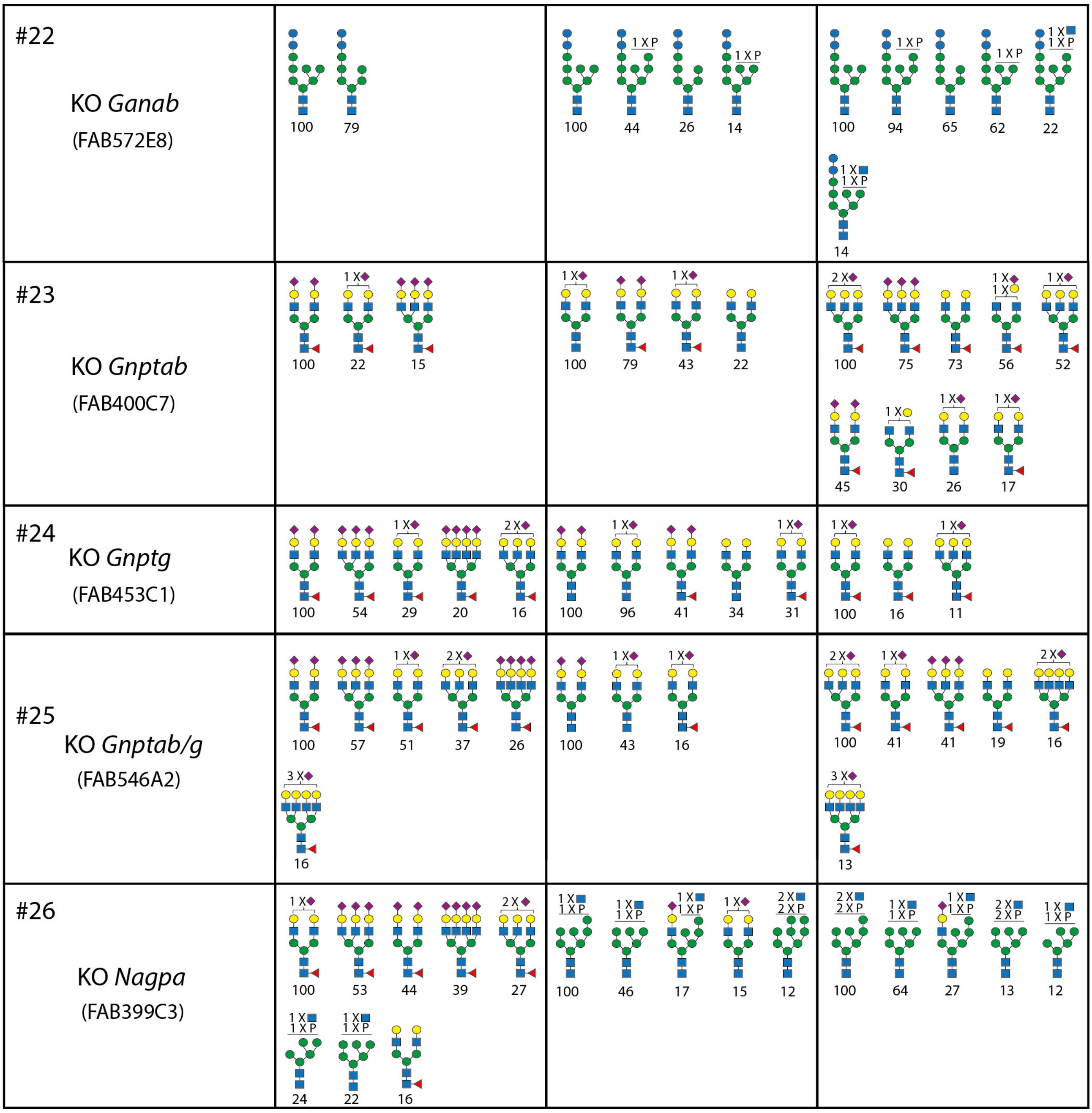

Supplementary Figure 2 continued.

(Page 5 of 12, continued)

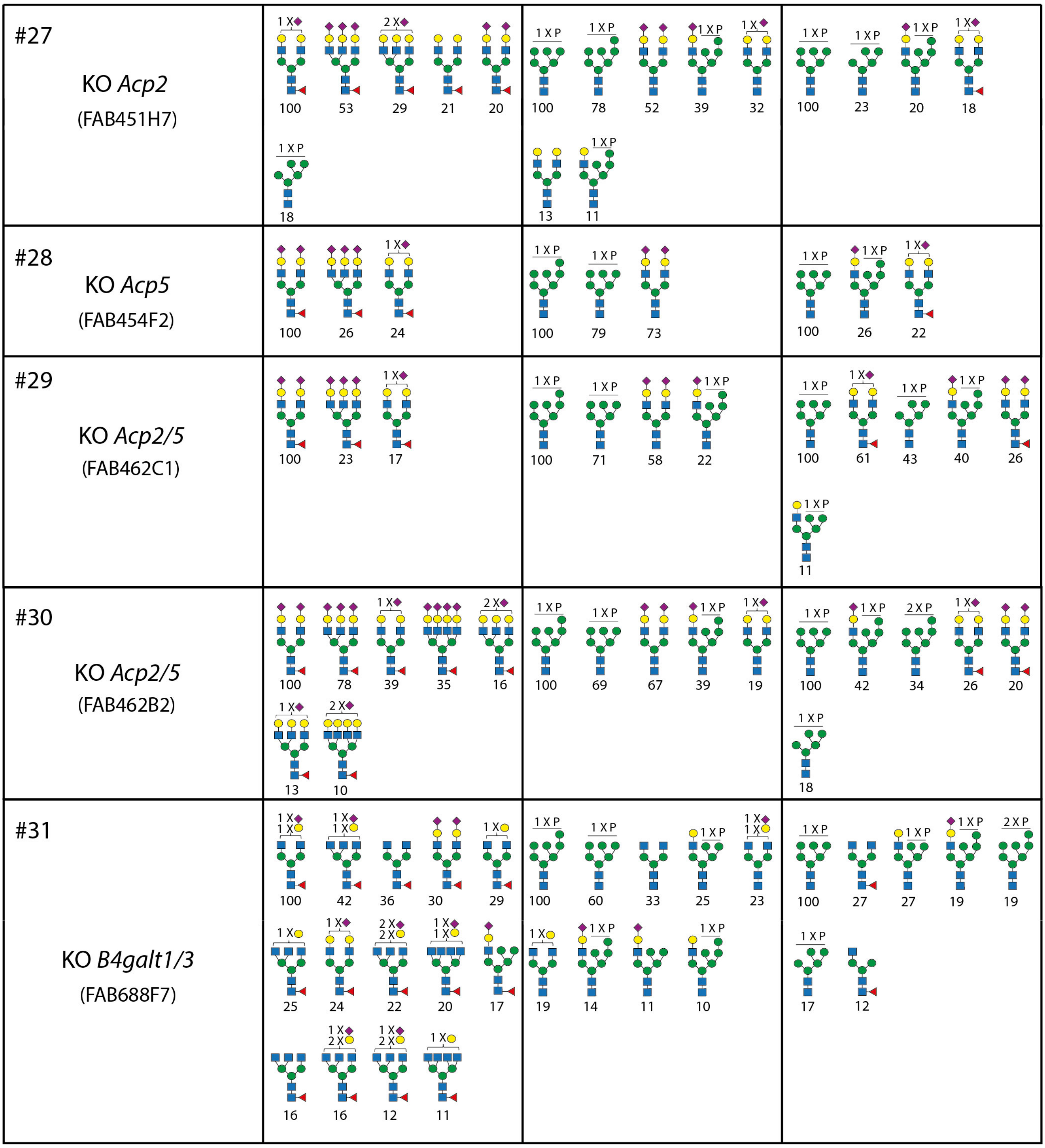

Supplementary Figure 2 continued.

(Page 6 of 12, continued)

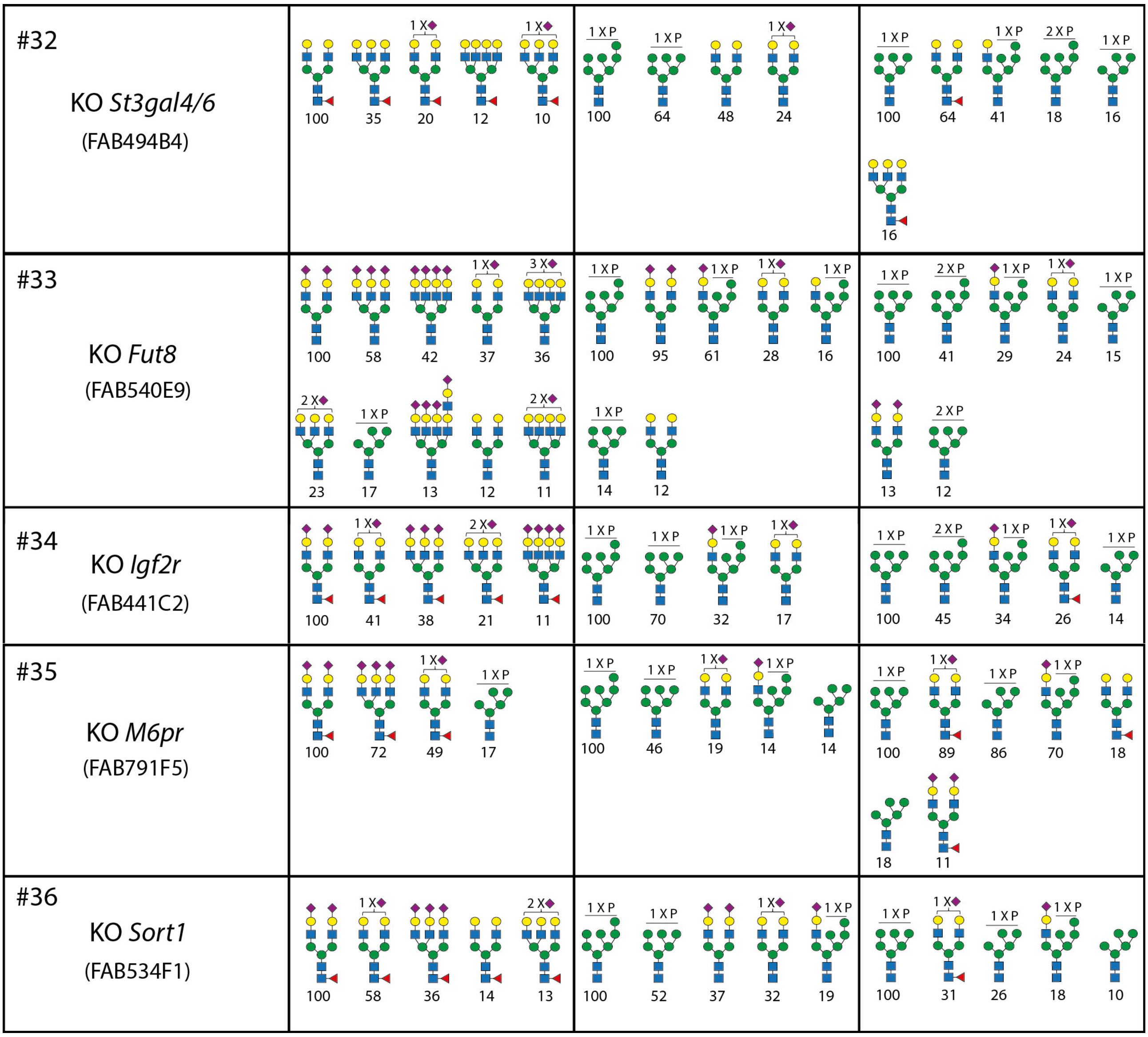

Supplementary Figure 2 continued.

(Page 7 of 12, continued)

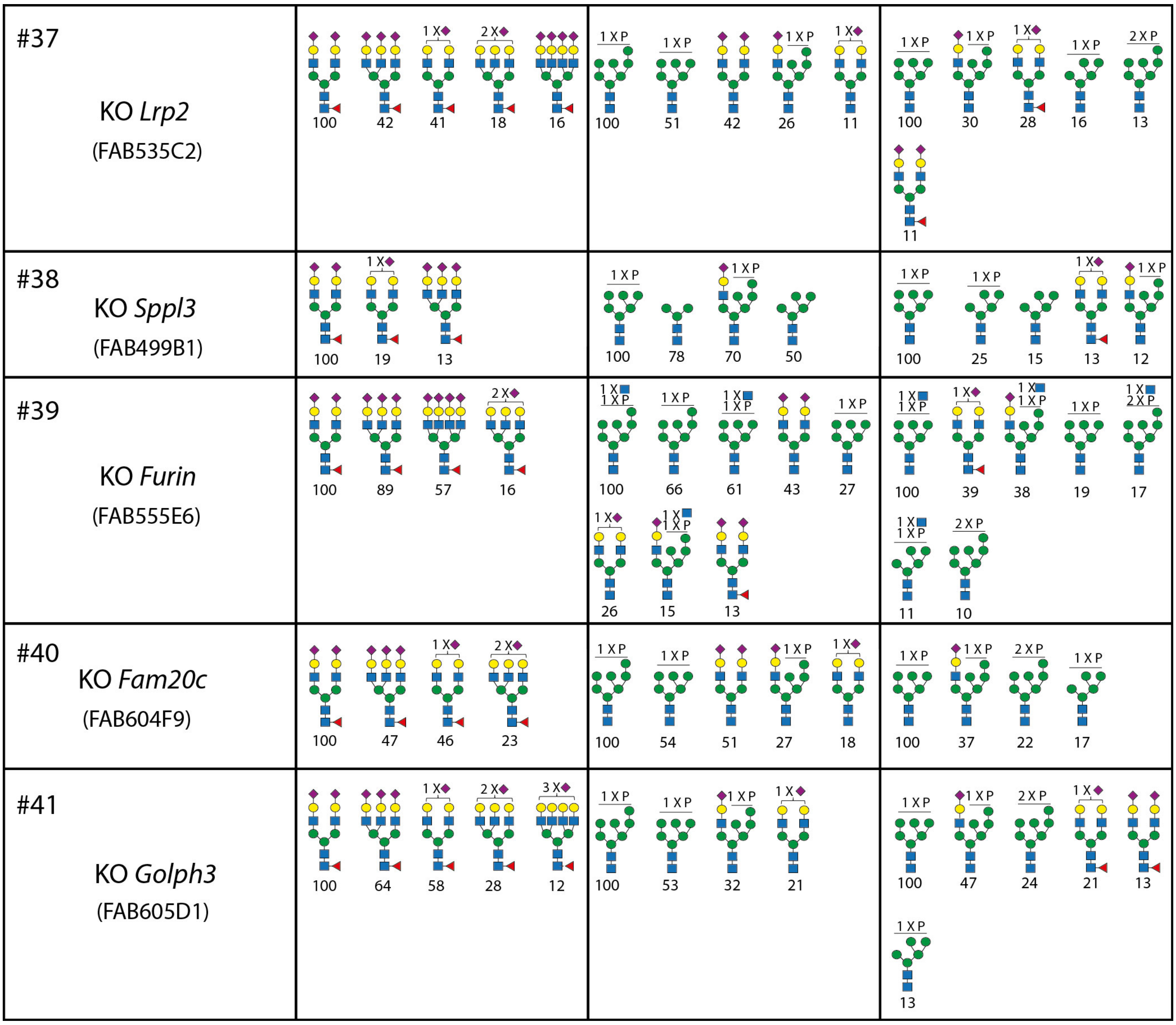

Supplementary Figure 2 continued.

(Page 8 of 12, continued)

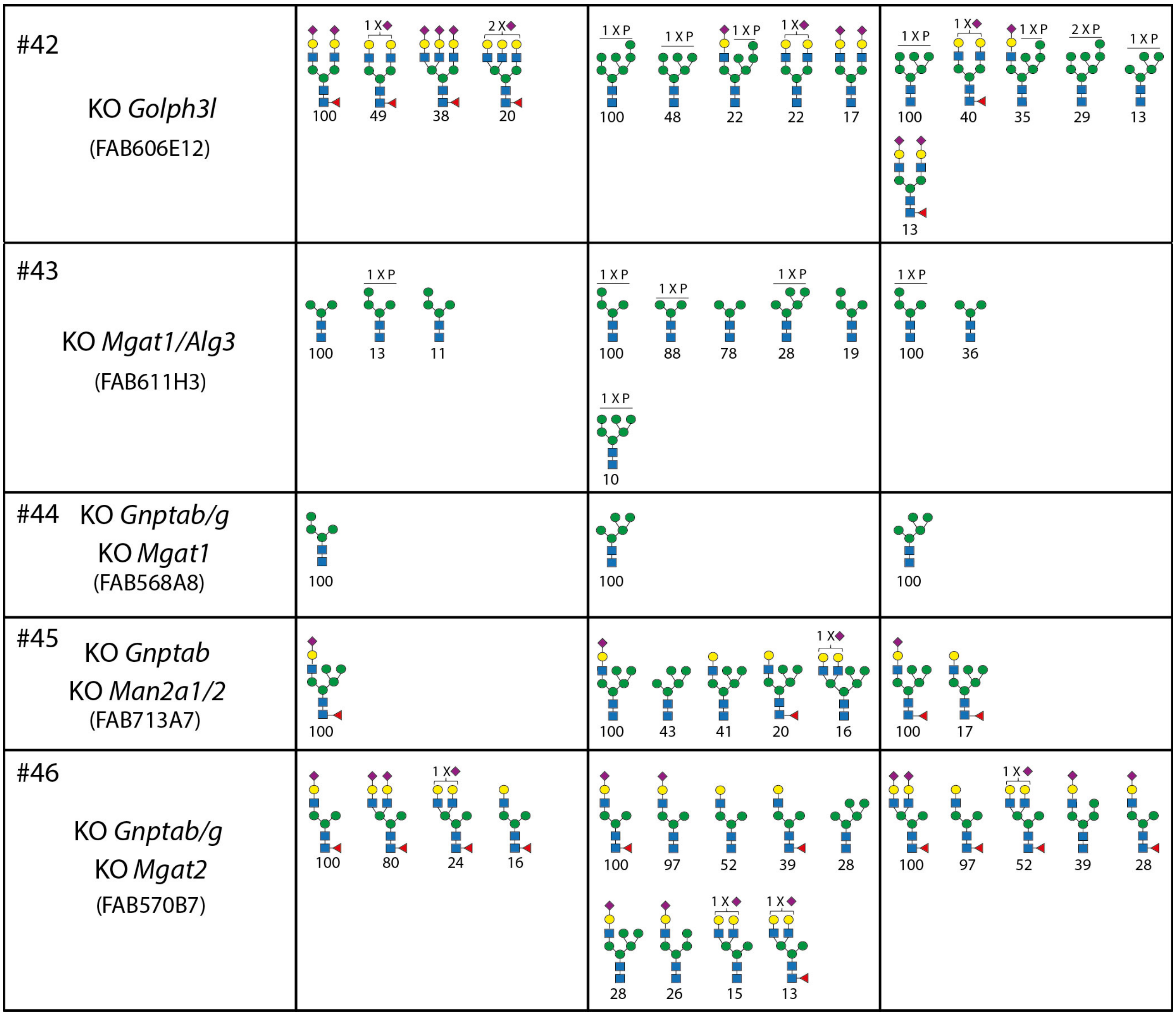

Supplementary Figure 2 continued.

(Page 9 of 12, continued)

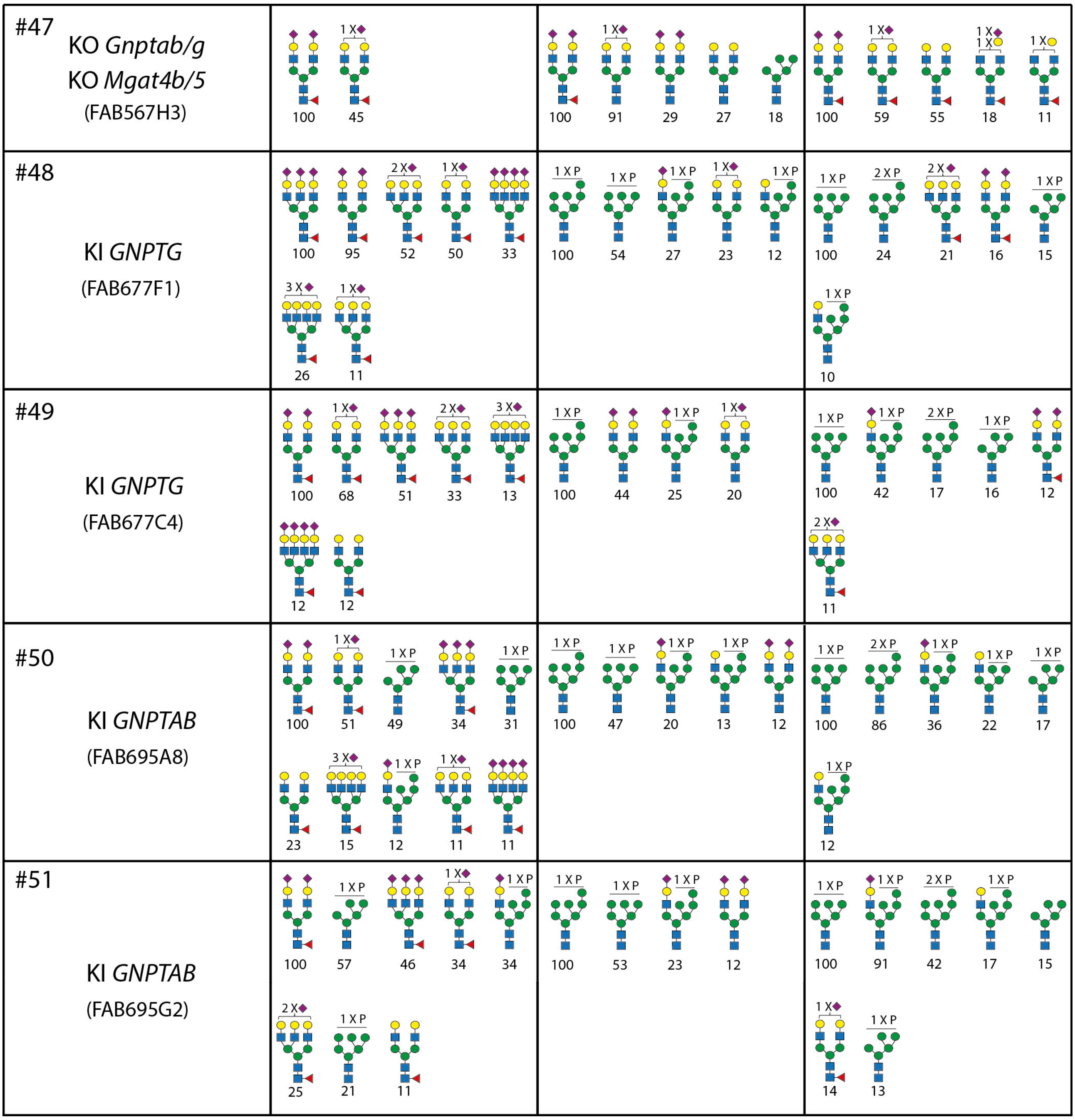

Supplementary Figure 2 continued.

(Page 10 of 12, continued)

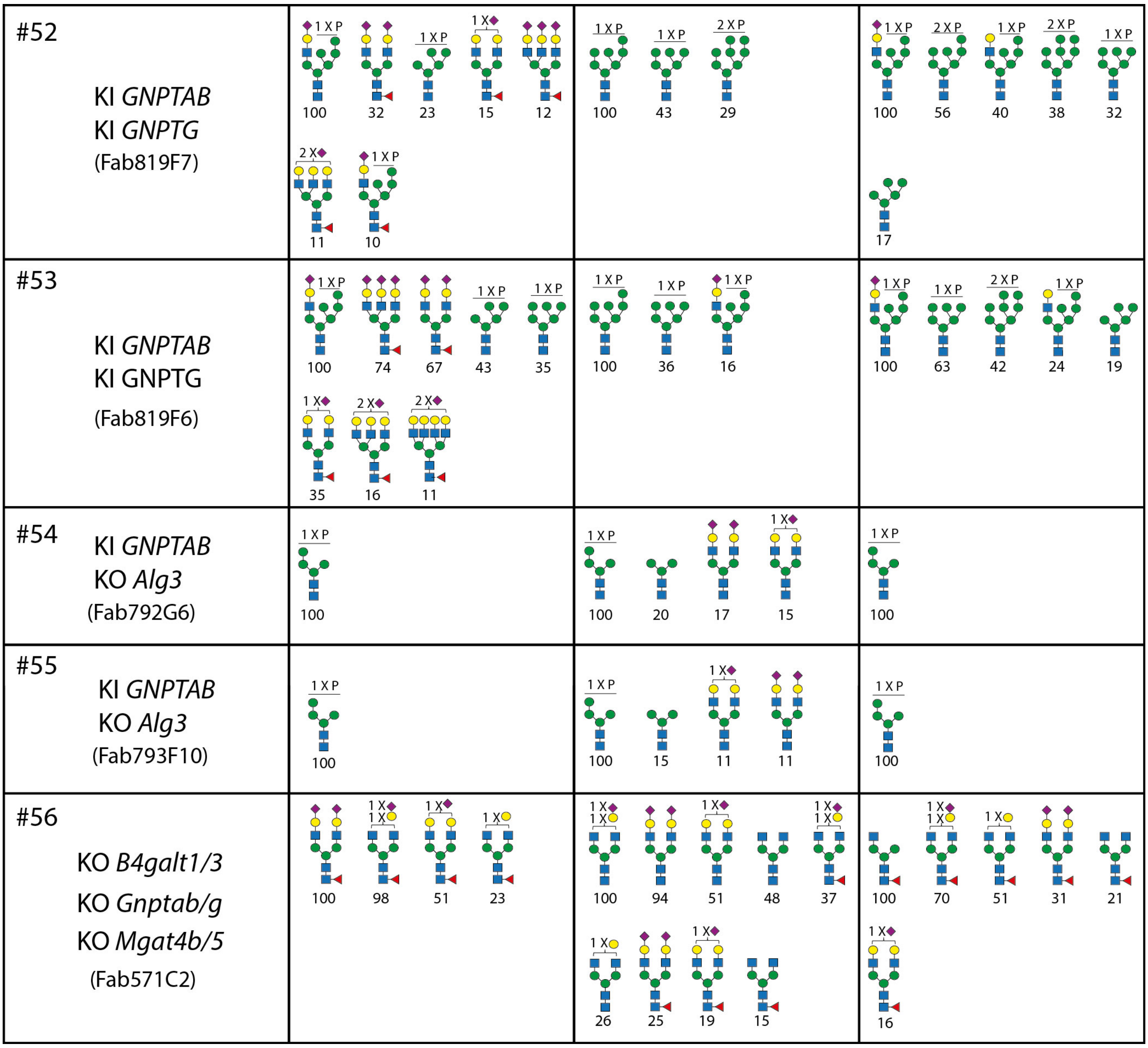

Supplementary Figure 2 continued.

(Page 11 of 12, continued)

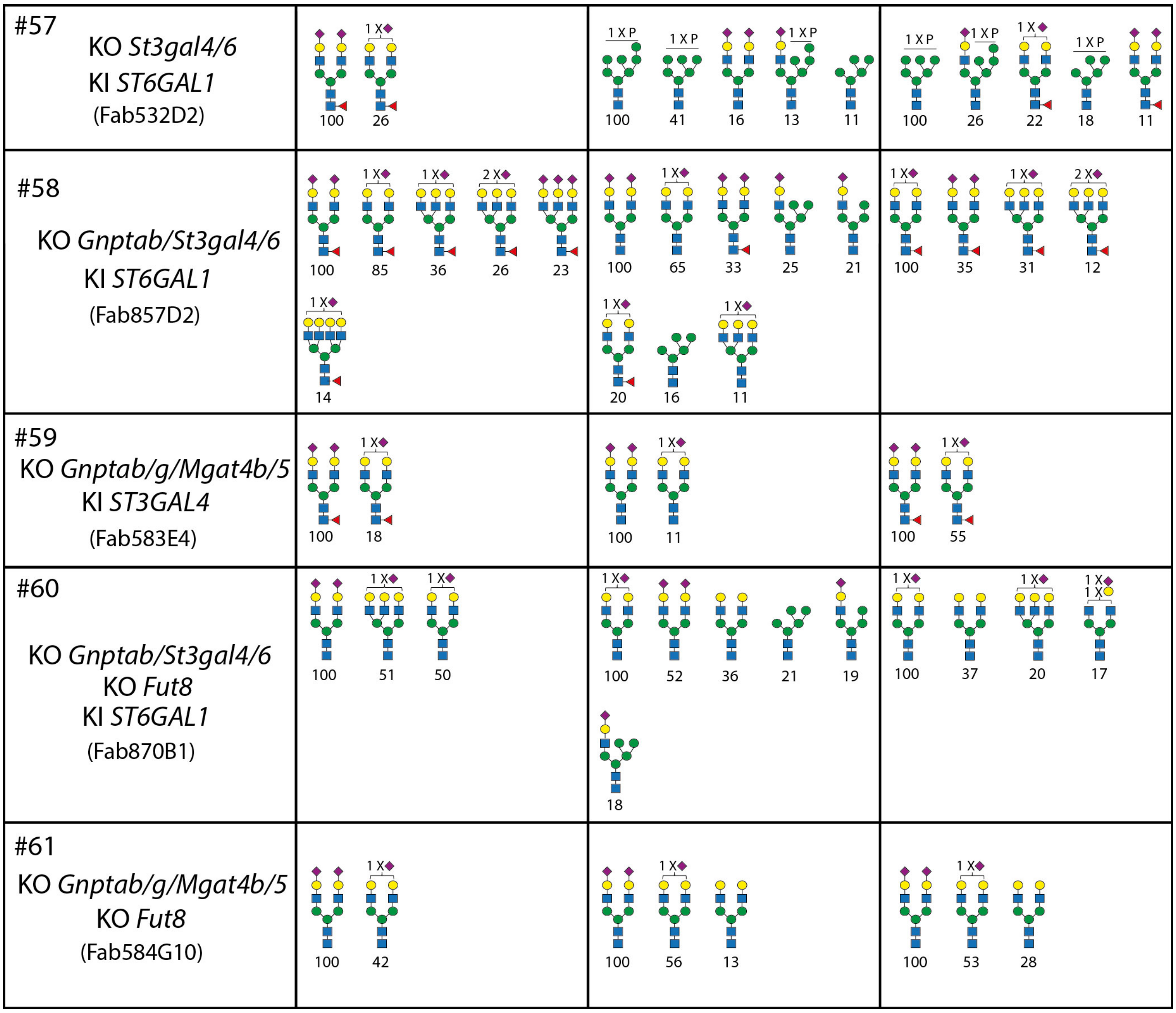

Supplementary Figure 2 continued.

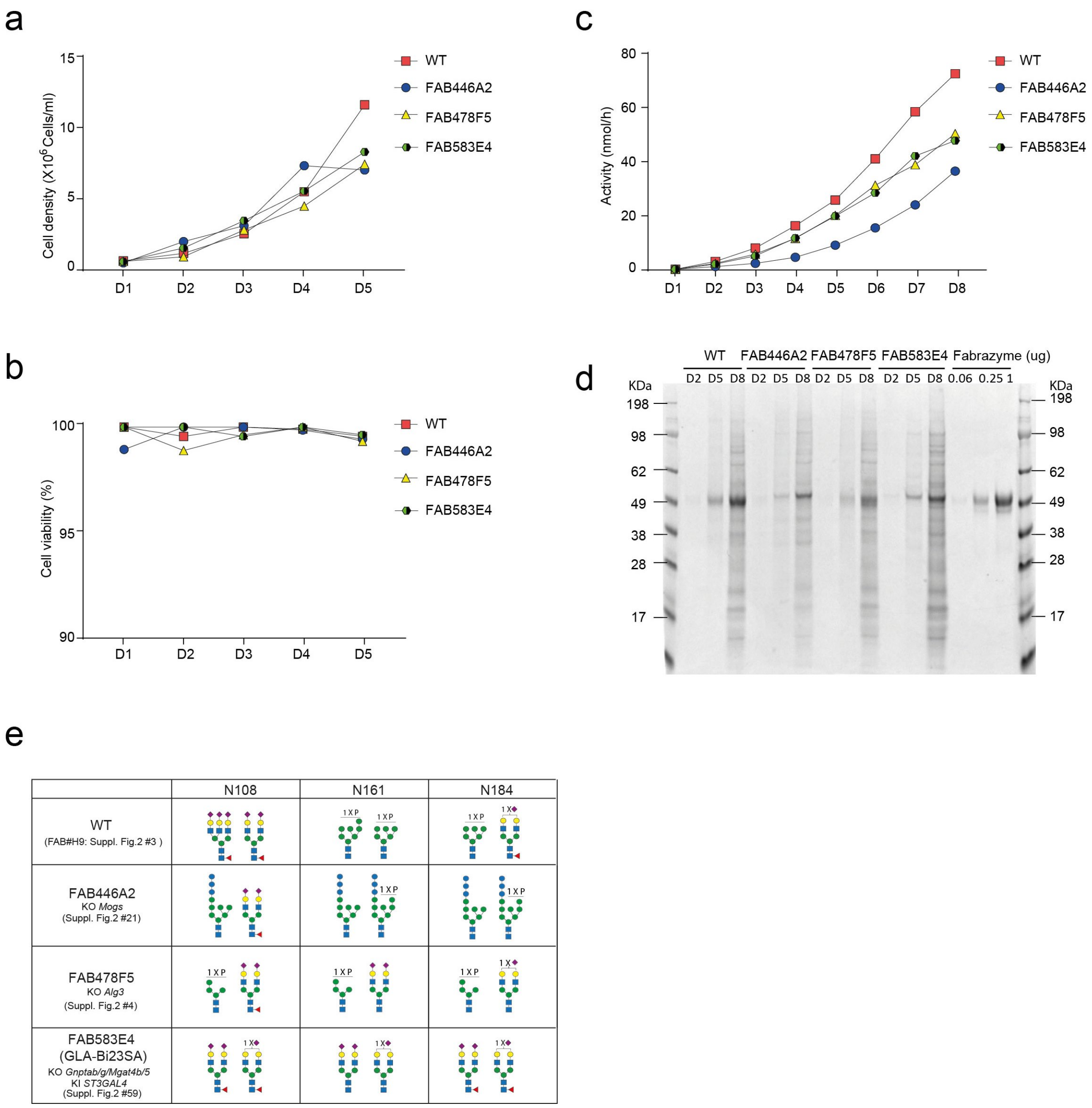

Supplementary Figure 3. Characterization of growth and yield performance of glycoengineered CHO clones expressing GLA.

a Viable cell density and **b** cell viability as determined by Trypan blue exclusion test. Data from day 1-5 are shown as accurate cell counting after day 6 was complicated by tendency for clumping of cells. **c** Yield of GLA enzyme activity determined in culture medium (2.5 μL) by release of p-nitrophenol per hour with a pNP-Gal enzyme assay. The substrate concentration was reduced to 1.2mM to fit the linear regression of absorbance at 405nm. **d** SDS-PAGE Coomassie analysis of GLA in culture medium (10 μL loaded) after two, five and eight days of culture (D2, D5 and D8, respectively). **e** Summary of the glycan features of the glycoengineered CHO clones and gene modification information. The two most abundant N-glycans are illustrated and detailed structures shown in **Supplementary Fig. 2, Panels 3, 21, 4 and 59**). Source data are provided as a Source Data file.

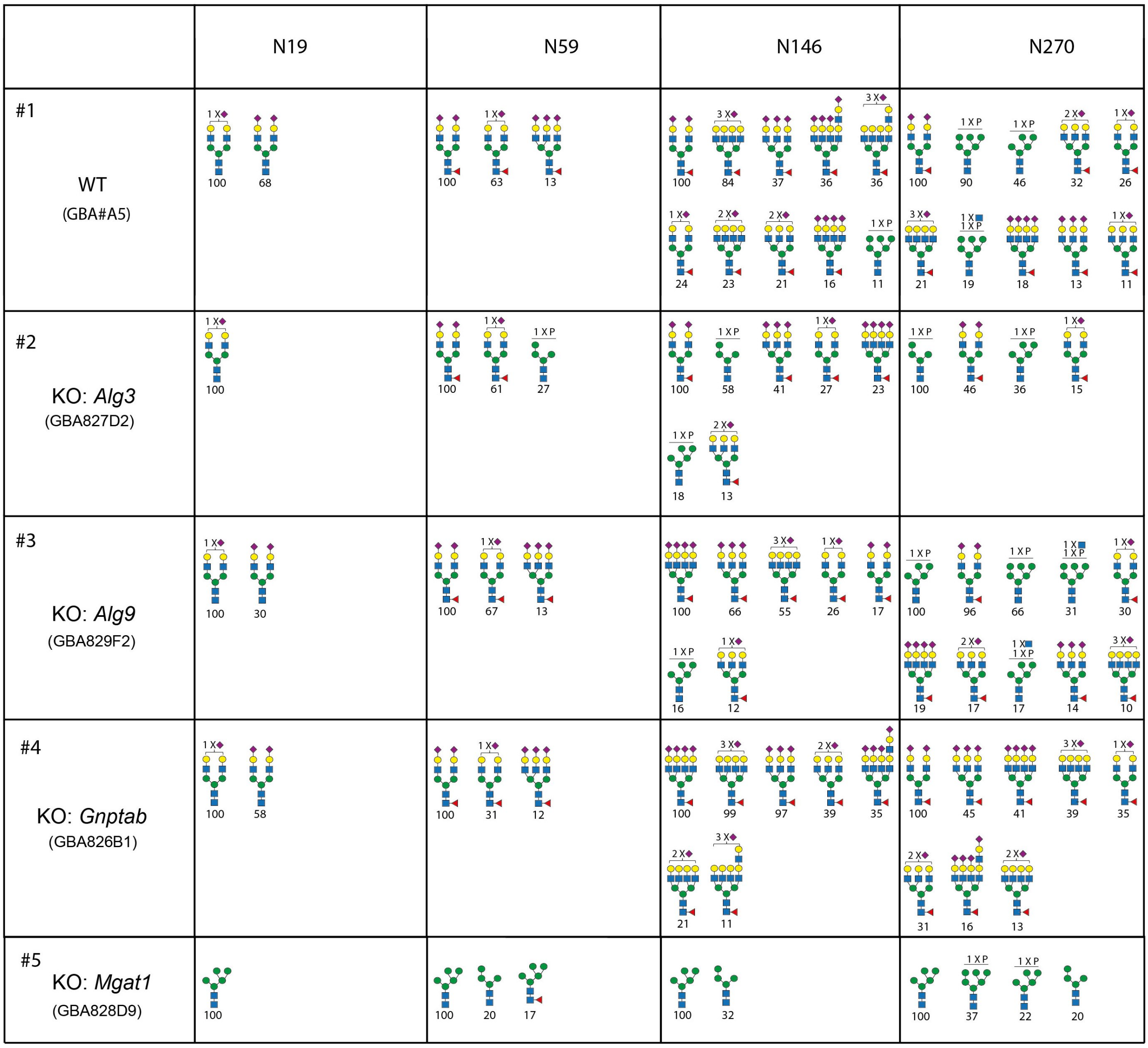

Supplementary Figure 4. Detailed presentation of N-glycans identified by site-specific N-glycan profiling of GBA produced in CHO WT and engineered KO clones as indicated.

N-glycan structures and their relative abundances at each of the four N-glycosites (N19, N59, N146, and N270) of GBA are illustrated with their relative abundance adjusted to the most abundant structure. Minor glycoforms identified with relative abundance less than 10% are not shown. Same N-glycan composition may represent isobaric structures.

(Page 1 of 2, continued)

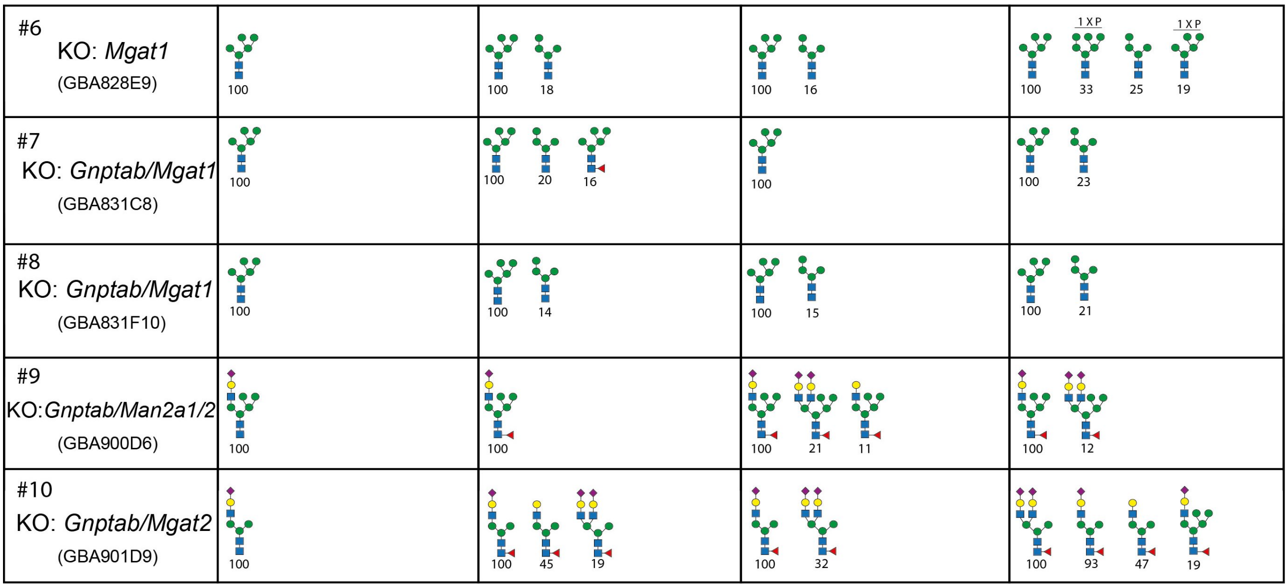

Supplementary Figure 4 continued.

a

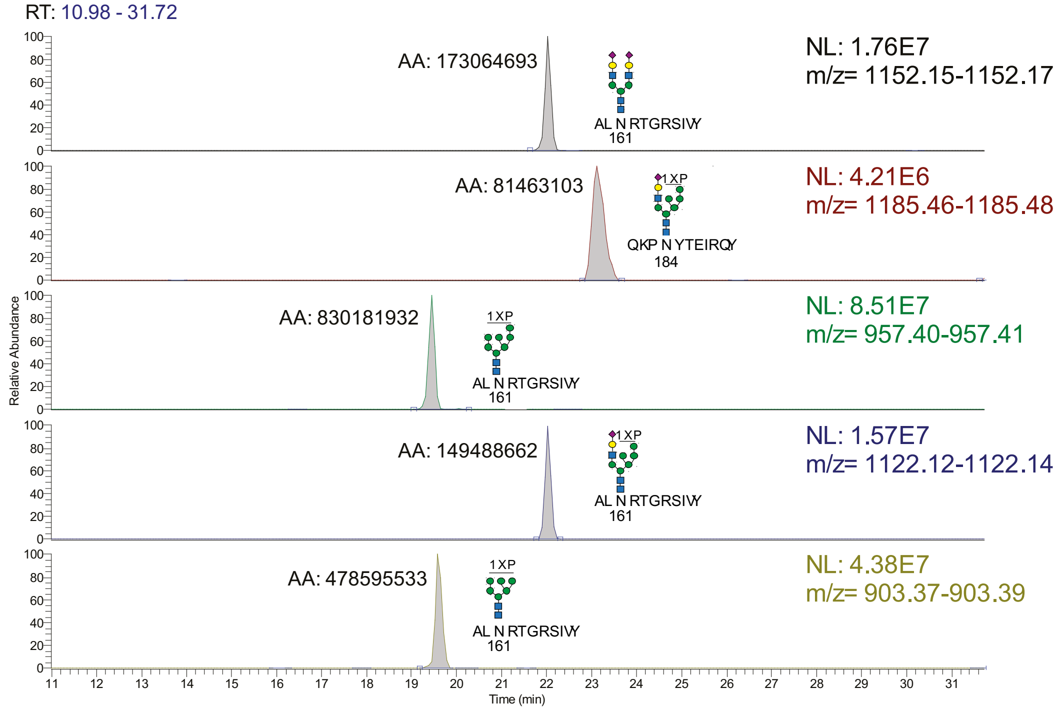

b

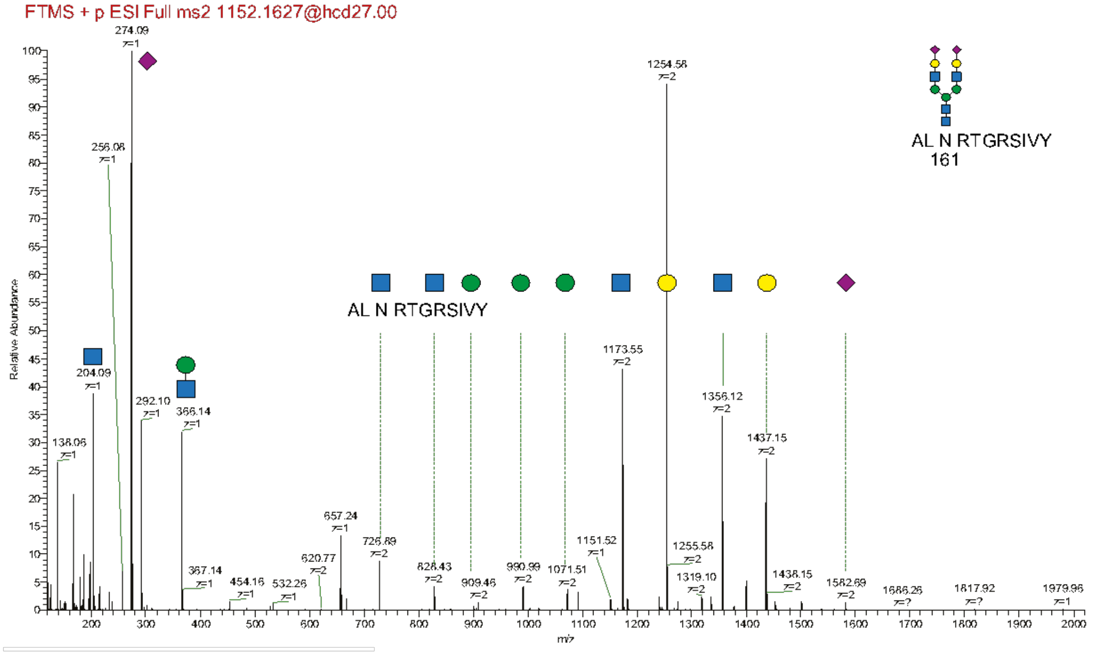

Supplementary Figure 5. Targeted MS/MS manual annotation of the most representative glycopeptides.

a Extracted ion chromatograms of 5 representative glycopeptide precursors of GLA produced in CHO WT. **b-f** MS2 manual annotation of these precursors.

(Page 1 of 3, continued)

c

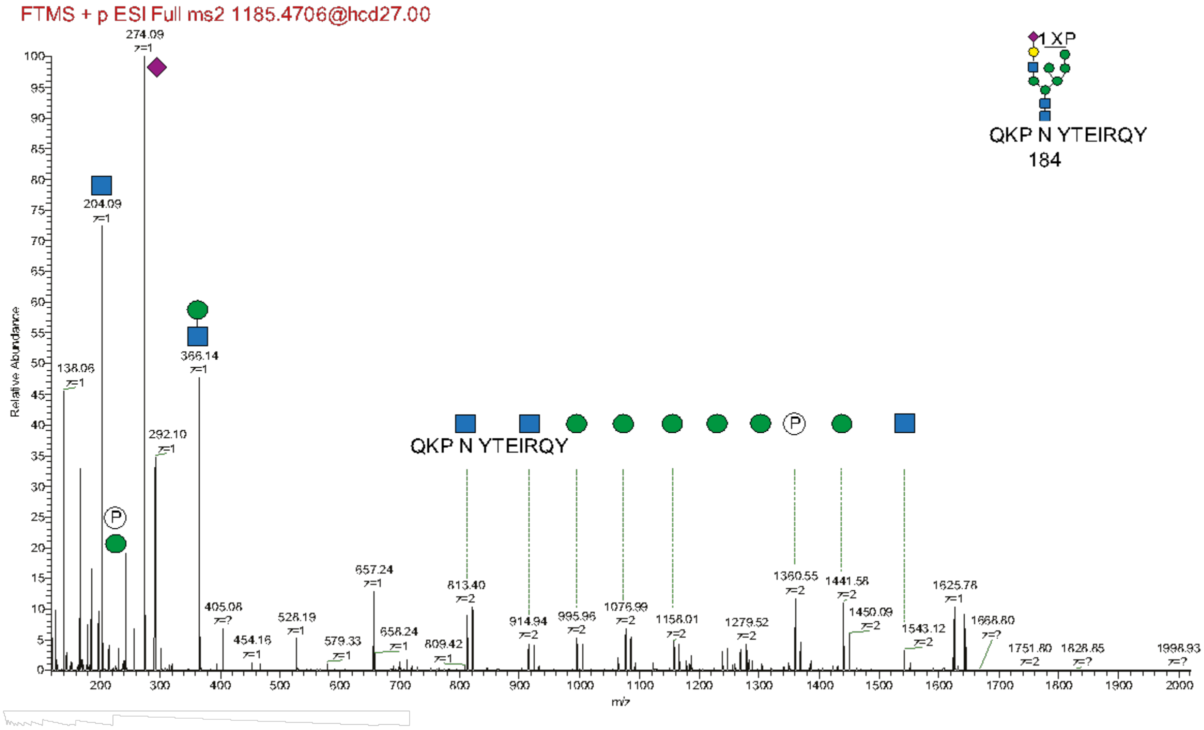

d

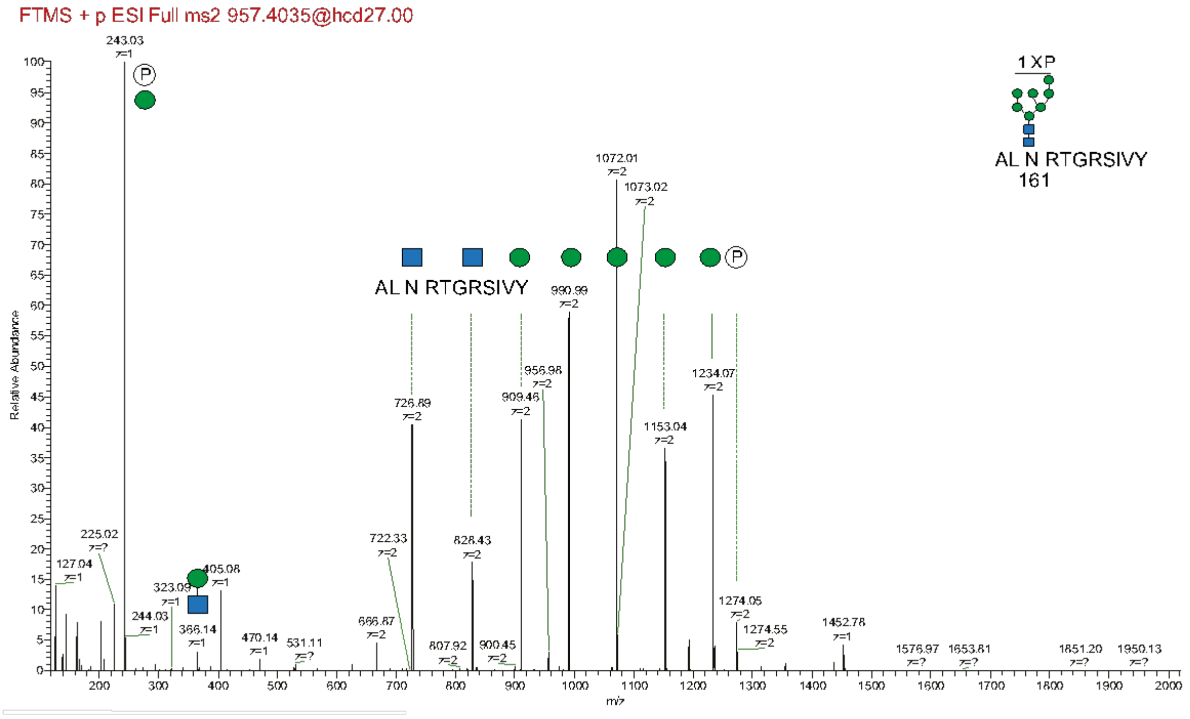

Supplementary Figure 5 continued. (Page 2 of 3, continued)

e

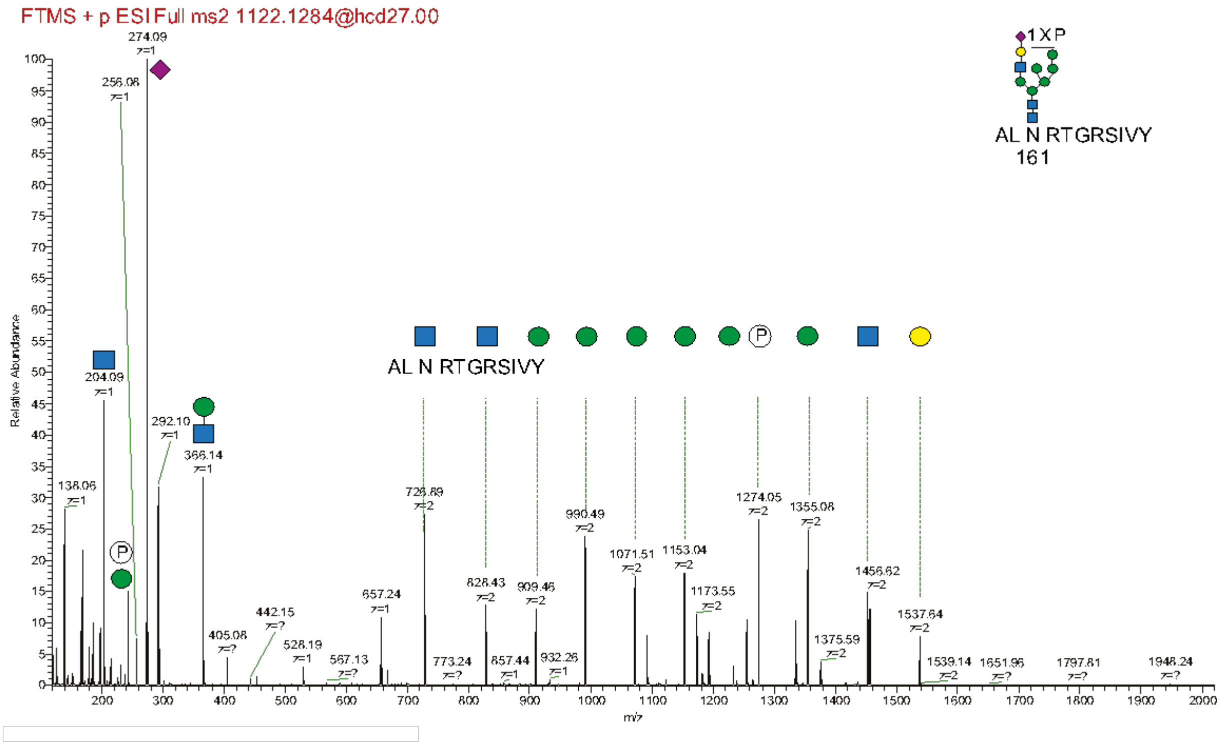

f

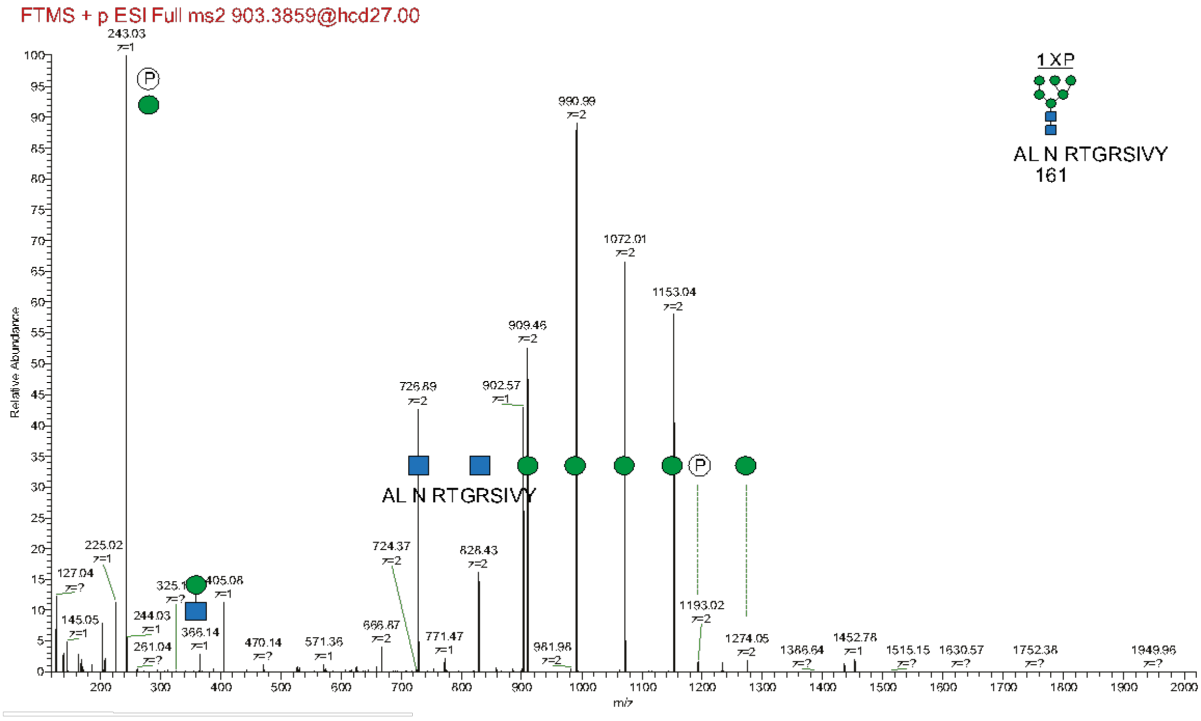

Supplementary Figure 5 continued.

(Page 3 of 3)

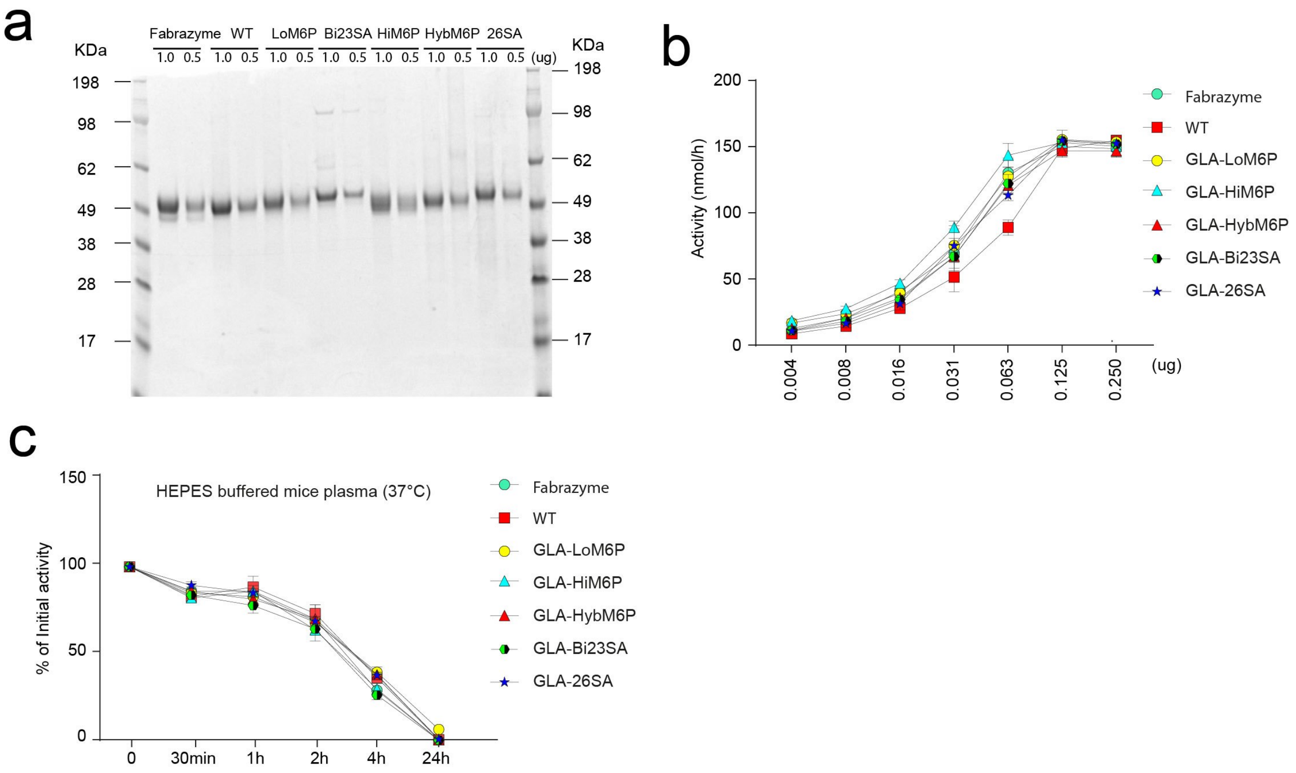

Supplementary Figure 6. In vitro assay of enzyme specific activity and plasma stability of GLA glycovariants.

a SDS-PAGE Coomassie analysis of the purified GLA variants used in mouse studies. **b** Specific activity of GLA variants assessed by a pNP-Gal enzyme assay. Release of p-nitrophenol per hour was used to show the enzyme activity. Each data point represents the mean of three replicates ± Standard Deviation (SD). **c** In vitro stability of GLA variants (10 µg/ml in HEPES buffered mouse plasma) at 37°C was measured by the remaining activity. Each data point represents the mean of three replicates ± SD. Source data are provided as a Source Data file.

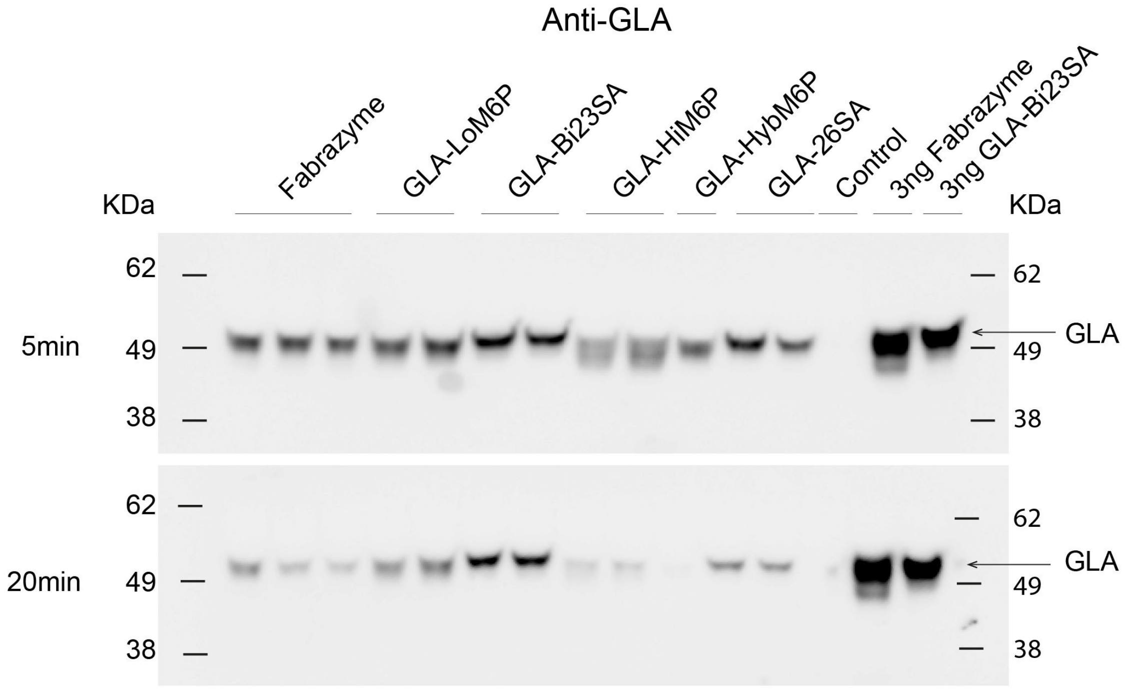

Supplementary Figure 7. SDS-PAGE Western blot analysis of infused GLA glycovariants in mouse plasma.

Plasma from mice infused with 1 mg/kg GLA variants used in Figure 5c collected at 5 and 20 min time points were analyzed. Blood samples were collected from tail vein bleeds, and plasma separated by centrifugation and diluted 100-fold in 0.1% BSA. Each lane represent one randomly selected mouse from the indicated group, and 10 µl of the diluted plasma was loaded in each lane. Control indicates was plasma collected from an untreated Fabry mouse. 3 ng Fabrazyme and GLA-Bi23SA was loaded respectively as positive controls. A rabbit anti-GLA polyclonal antibody (Sigma) was used 1:1,000 and an HRP-conjugated goat anti-rabbit immunoglobulins 1:3,000 (Dako P0448). Source data are provided as a Source Data file.

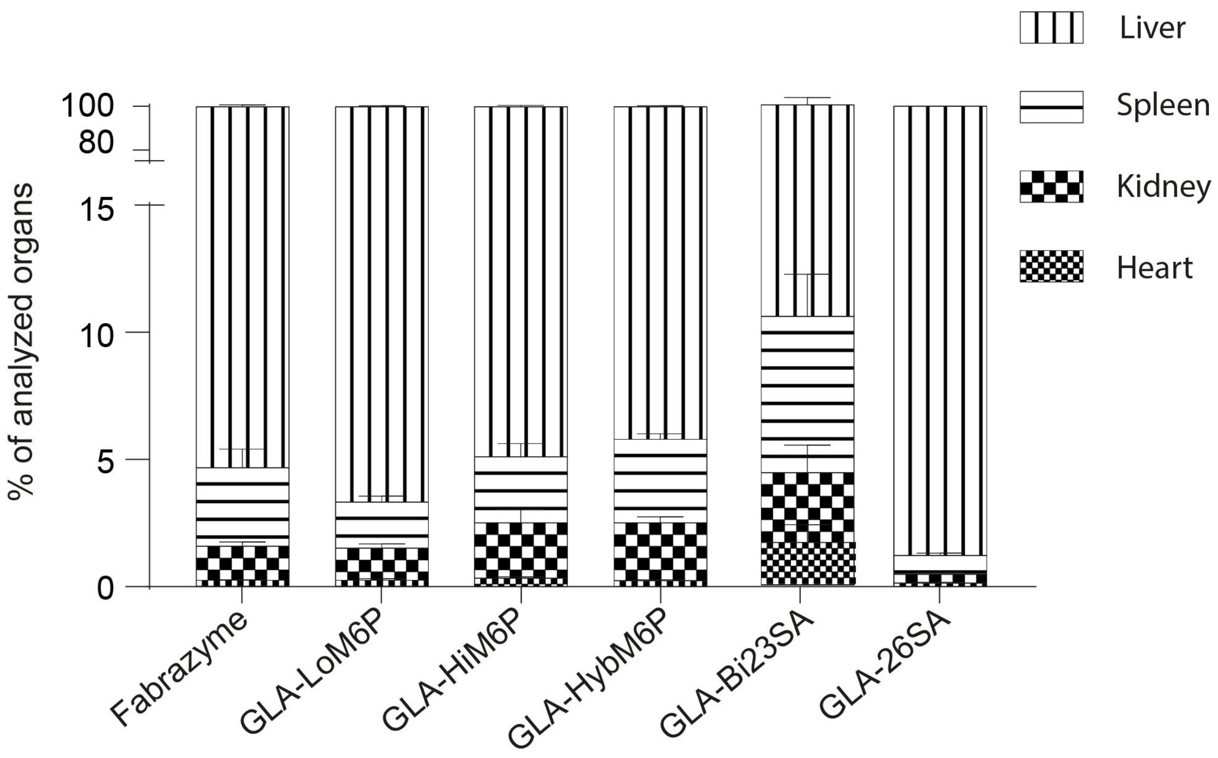

Supplementary Figure 8. Relative distribution of GLA glycovariants among the four major visceral organs.

GLA enzyme activity in dissected organs was assayed 24 h after 1 mg/kg infusion (same group as Figure 5e, Exp. #1). Activities in each whole organ and the sum of total recovered activities in the four major visceral organs were calculated. Relative distribution was shown by the % of each organ among the sum of total recovered activities. Error bars are presented with SD. Source data are provided as a Source Data file.

Supplementary Table 1. CRISPR gRNA design and list of PCR primers used for gRNA target sites.

Gene	gRNA	Forward primer (5'-3')	Reverse primer (5'-3')
Acp2	GCTCTGCGGCAGCGCTATAG	TCGTCTCTTCCCAGACAAGC	TAGGGTCTGTGAGCCATCCC
Acp5	GGATGCACGGACGGTACTGC	GTGCAGTTTTCAGGGGCTTG	CTCCCCAGAGTAAGGTCCCA
Alg1	TTCTGCAAGAGCTCATCTCG	CCCCAGTACACAACTACCCC	AGTACATGCTGGCCTTGAACA
Alg2	CTGTGACGTGAAGATATGGA	CTGCTGCTGGACAGTTCCAA	ATTGCAGAAGCTCGAGCGAA
Alg3	GCTGCTGGGCTGCGGAAACG	TAGCTAGAAACCCTGGTGCC	TAGTGAACTCACATGCCACCC
Alg5	GGACTCTAAGTTCACTTACG	TAGTAGGAGAGAGCCGACCC	CTTGGGTTCCTCCAGCAAGT
Alg6	TCTTAATAGGACTCACAGTG	AAGCAGATGCAGCCCACTCA	CAAGTGACGGACTTAGCAGGA
Alg8	TCGGTGTACTTCAAAATCCG	GTGCAGTGGTCTAAGAACCCA	TCAAGGCCTGGCAGCTTAC
Alg9	GAGCAGACATTTGAAAGCAG	GCCCAAGACCATCGGTTAGAT	TGTCCGGATTTAGTCTTCGCT
Alg11	ACTGGTGACATTAATGTCAG	TGAGTCCCTTTCTTTTTGTGCC	TCAGGAACACGCTGTGTCAG
Alg12	AAATCACCAGGCAAGTCAGG	CAGTGTGACCTTAAGCAGGGT	CAGGTCATGCGTAGCCTGTA
Alg13	GATCTTGTCATCAGCCACGC	AGTTATGAACCACGGAGCCA	TTGGAAGCTTAGCCAACTGGT
Alg14	CTGCGGCAGCTAGAATCAGG	TTTGACCGCCCAACTCATCA	AGCGCTCGTAAAGGTGCTAA
B4galt1	GGGCGGTCGTTATTCCCCCA	CCCAAACCTCACCTGGTTGAT	GCTGGCTAACATCTTCGTTCC
B4galt3	GCAGGACGGTACCGGCCCCC	ATGCCATATGCAAGCTGCTG	GTGGGTCCTGTGTCGGTATC
Fam20c	GGGAAGCCTGACCAGATCGA	ATAGGTCACCGACTCTCCCT	GCCAATAACATCTGCTTCTACGG
Furin	GACCAAGCGGGACGTGTATC	GCCCATCTCGGTCTCATTGC	TGGGGAAGAAGACCAGAACCC
Fut8	GATCCGTCCACAACCTTGGC	AGAGTCCATGGTGATCCTGC	TACTGTTTAAGGGGAGGGGGA
Ganab	GAAGGCTTCGATCCTCTAGC	GTCGTCTTGCCAACCCCAAA	CACACCCAGTCTCTTCCCAA
Gnptab	GTCACATTCATCGCATCGAG	ACCAACGGGCAGATTCCTTC	CTAGGTGCCCACCCATCTTAG
Gnptg	GCGATGGCGGTGCGGGTGGC	CTTCCGGTTTTGAGCGCAG	CAGCCAAGGGCTTTCCTCG
Golph3	GAAAGGCTCAGTGCAACACT	GCACAACTGACTCCAGGATG	GAGCTCTTCAGATGCCATAACC
Golph3l	TGACTTCAGTTCGACGGGTA	CTCTTTCCCATGTTCCTCCA	TGTGTGTGTATAGGTCTTCTGTGG
Igf2r	GACAAAAACCTGTCGATCAG	GCTACACATGGGAGGCTGTT	CAAACCCAAAGCTGCGGAAA
Lrp2	TCACACAAGGAATTCCAGTG	ATCAGTGCCCACTGCCTAAC	AAGGAACCCAGGTCAAGCAA
M6Pr	GCTATAGATTCAGAGTATGC	AAGGGAGGGGTGCAGTTTTT	GACCAGCTGTGGAACTAGGC
Man1a1	GTAAATATACGCTTCGTCGG	TGGGCAAGCACACAGGTTTA	TGACCACCGGAACACGAAAA
Man1a2	GTCTGTGTTCGTCGGGTCCA	CCACAGGGCTACCTTGAGAC	GTCCTTCCAGGCTATGGCTC
Man1b1	GAGTACATACCACCTATCGG	AGCACCAGCACAAAGGGATT	GCTTTCACCCTCTCATTACGC
Man1c1	GCCCCGGGCGAGGACGATCC	TGGAGGTGATGGCCGAAAAC	CAGTGTGCCTGAAGGGTCTC
Man2a1	GAGTGAAGCCTCGATCGGGT	TAATCACAGCTGCGAGGTGG	ACTGCTATGCACCCCCATTC
Man2a2	GCCCAGAGAAAGCGTCGTCG	AGCGGCATATTCAGGGGAAC	GGGACTGCATACATTGGCCT
Manea	ATAGCCAAGAACTATCCACA	CGCCCCCTTGGAAAACAAA	CGAACTAATTACCAACCAATTGAGG
Mgat1	GAGGGGGTCGCAGGCACACG	GTGCTTTGGGGTGCTATCCT	TGTGACTGCACTGCCATAGG
Mgat2	GCGACCGGTACCGCAGCGTT	GCGACAAAGGAAGAACGACG	TAGGTCTCTGGGGCAGTCTC
Mgat4b	GAGAGGCAGGCGCTGCGGGA	TAGCCTGTGTGTGTCAACCC	TGGGGAAGGGACAGGTTAGA
Mgat5	GACAATCTCGTCAATGGCAC	ACCTGCAGAGGTTTTCAGTTCT	GCCTTCACAACAATCATGCCA
Mogs	GGTGTCCCTGTTCTTCTACG	TTTAGCTCAGCCCACTCCAG	CTCCCTACCCGTACCACTCT
Nagpa	GGGCTGCAGAACGCGCAGTT	AGAACGGTGGTTTCTTCCGC	GCGTTCAATGACACACGACT
Sort1	TTAACAGCAGAGGTATCTGG	AGGACCATGCCCTGCTCTC	ATAGCCAGATGGGGACAGGTAG
Sppl3	GAGGCTTGGCAGGCGGACAA	ATGTCACCGACAAACGGGAC	CCACACACCAACTGATCCCC
St3gal4	GGTCGAAGTGGGCCGACTCA	AAGAGCGTGTCTGGGTTGTT	GCAGGGTCCACTTCTGGATT
St3gal6	GGAGTTGTGATCATTGTGAG	TCTTGGGTGCTTCTGAGTGTG	GGACACAGAAAATGGGATGTTG

Supplementary Table 2. Summary of CHO mutant clones stably expressing GLA and cell line ancestry.

Project number	Targeted genes	Parental CHO line
FAB399	KO *Nagpa*	WT#H9*
FAB400	KO *Gnptab*	WT#H9
FAB441	KO *Igf2r*	WT#H9
FAB442	KO *Man1a1*	WT#H9
FAB443	KO *Man1a2*	WT#H9
FAB444	KO *Man1b1*	WT#H9
FAB445	KO *Man1c1*	WT#H9
FAB446	KO *Mogs*	WT#H9
FAB451	KO *Acp2*	WT#H9
FAB453	KO *Gnptg*	WT#H9
FAB454	KO *Acp5*	WT#H9
FAB462	KO *Acp2/5*	WT#H9
FAB478	KO *Alg3*	WT#H9
FAB479	KO *Alg6*	WT#H9
FAB480	KO *Alg9*	WT#H9
FAB494	KO *St3gal4/6*	WT#H9
FAB495	KO *Mgat1*	WT#H9
FAB496	KO *Mgat2*	WT#H9
FAB497	KO *Mgat4b/5*	WT#H9
FAB499	KO *Sppl3*	WT#H9
FAB532	KI *ST6GAL1*/KO *St3gal4/6*	FAB494 B4
FAB534	KO *Sort1*	WT#H9
FAB535	KO *Lrp2*	WT#H9
FAB540	KO *Fut8*	WT#H9
FAB546	KO *Gnptab/g*	WT#H9
FAB555	KO *Furin*	WT#H9
FAB560	KO *Manea*	WT#H9
FAB567	KO *Mgat4b/5/Gnptab/g*	FAB546A2
FAB568	KO *Mgat1/Gnptab/g*	FAB546A2
FAB570	KO *Mgat2/Gnptab/g*	FAB546A2
FAB571	KO *B4galt1/3/Mgat4b/5/Gnptab/g*	FAB567H3
FAB572	KO *Ganab*	WT#H9
FAB583	KI *ST3GAL4/* KO*Mgat4b/5/Gnptab/g*	FAB567H3
FAB584	KO *Fut8/Mgat4b/5/Gnptab/g*	FAB567H3
FAB604	KO *Fam20c*	WT#H9
FAB605	KO *Golph3*	WT#H9
FAB606	KO *Golph3l*	WT#H9
FAB611	KO *Alg3/Mgat1*	FAB495B10
FAB662	KO *Alg8*	WT#H9
FAB664	KO *Alg12*	WT#H9
FAB667	KO *Alg5*	WT#H9
FAB677	KI *GNPTG*	WT#H9
FAB688	KO *B4galt1/3*	WT#H9
FAB695	KI *GNPTAB*	WT#H9
FAB712	KO *Man2a1/2*	WT#H9
FAB713	KO *Man2a1/2/Gnptab*	FAB400C7
FAB725	KO *Man1a1/1a2/1b1/1c1*	FAB442G8
FAB791	KO *M6pr*	WT#H9
FAB792	KI *GNPTAB* /KO *Alg3*	FAB695G2
FAB793	KI *GNPTAB* /KO *Alg3*	FAB695A8
FAB819	KI *GNPTAB/G*	FAB695G2
FAB857	KI *ST6GAL1*/KO *St3gal4/6/Gnptab*	FAB532D2
FAB870	KI *ST6GAL1*/KO *St3gal4/6/Gnptab/Fut8*	FAB857D2

* Clone WT#H9 was used as parental clone for all gene engineering.

Supplementary Table 3. Summary of CHO mutant clones stably expressing GBA and cell line ancestry.

**Project number**	**Targeted genes**	**Parental CHO line**
GBA826	KO *Gnptab*	GBA#A5
GBA827	KO *Alg3*	GBA#A5
GBA828	KO *Mgat1*	GBA#A5
GBA829	KO *Alg9*	GBA#A5
GBA831	KO *Gnptab/Mgat1*	GBA#A5
GBA900	KO *Gnptab/Man2a1/2*	GBA826B1
GBA901	KO *Gnptab/Mgat2*	GBA826B1

Supplementary Table 4. Sequence analysis of CHO mutant clones stably expressing GBA.

Clone	Targeted genes	InDels	Alignment
GBA826B1	KO *Gnptab*		
	WT		GTCACATTCATCGCATCGAGGGG
	KO	+1bp	GTCACATTCATCGCATCCGAGGGG
GBA827C2	KO *Alg3*		
	WT		GCTGCTGGGCTGCGGAAACGCGG
	KO	-1bp	GCTGCTGGGCTGCGGAAACGCGG
GBA827D2	KO *Alg3*		
	WT		GCTGCTGGGCTGCGGAAACGCGG
	KO	+1bp	GCTGCTGGGCTGCGGAACACGCGG
GBA828D9	KO *Mgat1*		
	WT		GAGGGGGTCGCAGGCACACGGGG
	KO	+1bp	GAGGGGGTCGCAGGCACCACGGGG
GBA828E9	KO *Mgat1*		
	WT		GAGGGGGTCGCAGGCACACGGGG
	KO	+1bp	GAGGGGGTCGCAGGCACCACGGGG
GBA829F2	KO *Alg9*		
	WT		GAGCAGACATTTGAAAGCAGTGG
	KO-alle1	-7bp	GAGCAGACATTTGAAAGCAGTGG
	KO-alle2	-2bp	GAGCAGACATTTGATTTGTGG
GBA831C8	KO *Mgat1*		
	WT		GAGGGGGTCGCAGGCACACGGGG
	KO	+1bp	GAGGGGGTCGCAGGCACCACGGGG
	KO *Gnptab*		
	WT		GTCACATTCATCGCATCGAGGGG
	KO	+1bp	GTCACATTCATCGCATCCGAGGGG
GBA831F10	KO *Mgat1*		
	WT		GAGGGGGTCGCAGGCACACGGGG
	KO	+1bp	GAGGGGGTCGCAGGCACCACGGGG
	KO *Gnptab*		
	WT		GTCACATTCATCGCATCGAGGGG
	KO	+1bp	GTCACATTCATCGCATCCGAGGGG
GBA900D6	KO *Man2a1*		
	WT		GAGTGAAGCCTCGATCGGGTTGG
	KO	-4bp	GAGTGAAGCCTCGATCGGGTTGG
	KO *Man2a2*		
	WT		GCCCAGAGAAAGCGTCGTCGAGG
	KO	-1bp	GCCCAGAGAAAGCGTCGTCGAGG
	KO *Gnptab*		
	WT		GTCACATTCATCGCATCGAGGGG
	KO	+1bp	GTCACATTCATCGCATCCGAGGGG
GBA901D9	KO *Mgat2*		
	WT		GCGACCGGTACCGCAGCGTTAGG
	KO	+1bp	GCGACCGGTACCGCAGCCGTTAGG
	KO *Gnptab*		
	WT		GTCACATTCATCGCATCGAGGGG
	KO	+1bp	GTCACATTCATCGCATCCGAGGGG

Note: Nucleic acids UNDERLINED are the gRNA targeting sequence;
Nucleic acids in RED are the PAM sequence;
Nucleic acids in GREEN are insertions;
Nucleic acids in GRY are deletions.
