# Supplementary material for: The glycosylation design space for recombinant lysosomal replacement enzymes produced in CHO cells
Source: Nat Commun. 2019 Apr 30;10:1785. doi: 10.1038/s41467-019-09809-3 (PMC6491494; doi:10.1038/s41467-019-09809-3)
Supplement: Supplementary file 3 — Description of Additional Supplementary Files [file 41467_2019_9809_MOESM3_ESM.pdf]

Description of Additional Supplementary Files

File Name: Supplementary Data 1
Description: Sequence analysis of CHO mutant clones stably expressing GLA was conducted with Sanger sequencing and aligned with the wild type genome sequence of CHO cells.
