# Supplementary material for: The glycosylation design space for recombinant lysosomal replacement enzymes produced in CHO cells
Source: Nat Commun. 2019 Apr 30;10:1785. doi: 10.1038/s41467-019-09809-3 (PMC6491494; doi:10.1038/s41467-019-09809-3)
Supplement: Supplementary file 5 — Reporting Summary [file 41467_2019_9809_MOESM5_ESM.pdf]

nature research

Corresponding author(s): Henrik Clausen

Last updated by author(s): Mar 22, 2019

Reporting Summary

Nature Research wishes to improve the reproducibility of the work that we publish. This form provides structure for consistency and transparency in reporting. For further information on Nature Research policies, see Authors & Referees and the Editorial Policy Checklist.

Statistics

For all statistical analyses, confirm that the following items are present in the figure legend, table legend, main text, or Methods section.

n/a	Confirmed	
☐	☒	The exact sample size (*n*) for each experimental group/condition, given as a discrete number and unit of measurement
☐	☒	A statement on whether measurements were taken from distinct samples or whether the same sample was measured repeatedly
☐	☒	The statistical test(s) used AND whether they are one- or two-sided<br>*Only common tests should be described solely by name; describe more complex techniques in the Methods section.*
☒	☐	A description of all covariates tested
☒	☐	A description of any assumptions or corrections, such as tests of normality and adjustment for multiple comparisons
☐	☒	A full description of the statistical parameters including central tendency (e.g. means) or other basic estimates (e.g. regression coefficient) AND variation (e.g. standard deviation) or associated estimates of uncertainty (e.g. confidence intervals)
☐	☒	For null hypothesis testing, the test statistic (e.g. *F*, *t*, *r*) with confidence intervals, effect sizes, degrees of freedom and *P* value noted<br>*Give P values as exact values whenever suitable.*
☒	☐	For Bayesian analysis, information on the choice of priors and Markov chain Monte Carlo settings
☒	☐	For hierarchical and complex designs, identification of the appropriate level for tests and full reporting of outcomes
☒	☐	Estimates of effect sizes (e.g. Cohen's *d*, Pearson's *r*), indicating how they were calculated

Our web collection on statistics for biologists contains articles on many of the points above.

Software and code

Policy information about availability of computer code

Data collection	SysBioWare software.<br>Vakhrushev, S., Dadimov, D. & Peter-Katalinic, J. Software platform for high-throughput glycomics. Analytical chemistry 81, 3252-3260 (2009)
Data analysis	SysBioWare software.<br>Vakhrushev, S., Dadimov, D. & Peter-Katalinic, J. Software platform for high-throughput glycomics. Analytical chemistry 81, 3252-3260 (2009)

For manuscripts utilizing custom algorithms or software that are central to the research but not yet described in published literature, software must be made available to editors/reviewers. We strongly encourage code deposition in a community repository (e.g. GitHub). See the Nature Research guidelines for submitting code & software for further information.

Data

Policy information about availability of data

All manuscripts must include a data availability statement. This statement should provide the following information, where applicable:

- Accession codes, unique identifiers, or web links for publicly available datasets
- A list of figures that have associated raw data
- A description of any restrictions on data availability

All mass spectrometry raw data underlying Figures 1, 2, 3, and 4 and supplementary Figures 2 and 4 have been deposited to the ProteomeXchange Consortium via the PRIDE partner repository with the dataset identifier PXD013140. Other data that support the findings of this study are available from the corresponding author upon request.

Field-specific reporting

Please select the one below that is the best fit for your research. If you are not sure, read the appropriate sections before making your selection.

☒ Life sciences ☐ Behavioural & social sciences ☐ Ecological, evolutionary & environmental sciences

For a reference copy of the document with all sections, see nature.com/documents/nr-reporting-summary-flat.pdf

Life sciences study design

All studies must disclose on these points even when the disclosure is negative.

Sample size

here were no statistical methods used to predetermine the sample size. For glycoengineering and site-specific glycopeptide analysis, multiple CHO clones (2-5) were obtained for each gene editing event, and we usually start with one clone for site-specific N-glycopeptide profiling until we see the necessity of repeating or chose the second clone. All the detailed N-glycopeptide profiling data are shown in supplementary information files, and all the raw MS data will be uploaded to the PRIDE archive upon acceptance. In our system, the gene modified clones are isogenic with each other, one clone is adequate to see the effect caused by gene modification.
For characterization of growth and yield performance of glycoengineered GLA expressing CHO clones, one clone from the most representative glycoengineered clones were chosen, and data was collected in a time course.
For in vivo study of pharmacokinetics, biodistribution and Gb3 clearance of different GLA glycovariants, 3-5 mice were used in each experimental group. The rational for this sample sizes is based on previous studies from our group and other groups with similar experimental setups which showed significance.

Data exclusions

No data were excluded from the analysis.

Replication

Some of the site-specific glycopeptide analysis were repeated independently with another gene modified clone, and these data are shown in supplemental information files. All attempts at replication were successful.
Animal studies were conducted blindly with sufficient sample size without replication.

Randomization

Mice were randomly assigned to each experimental group.

Blinding

Site-specific glycopeptide analysis were blinded without showing gene-modification information to the investigator. The mice studies to test the performance of different GLA variants were also blinded without showing the glycan strutures or the function of different glycans.

Reporting for specific materials, systems and methods

We require information from authors about some types of materials, experimental systems and methods used in many studies. Here, indicate whether each material, system or method listed is relevant to your study. If you are not sure if a list item applies to your research, read the appropriate section before selecting a response.

Materials & experimental systems

n/a	Involved in the study	
☐	☒	Antibodies
☐	☒	Eukaryotic cell lines
☒	☐	Palaeontology
☐	☒	Animals and other organisms
☒	☐	Human research participants
☒	☐	Clinical data

Methods

n/a	Involved in the study	
☒	☐	ChIP-seq
☒	☐	Flow cytometry
☒	☐	MRI-based neuroimaging

Antibodies

Antibodies used

Primary antibodies used: anti-His (C-term)-HRP (Invitrogen, P/N 46-0707, lot#1905395, 1:5000), anti-myc (clone 9E10, ATCC CRL-1729, undilued hybridoma curture supernatant), anti-GLA (Sigma, HPA000237, 1:300 dilution for IHC/ 1:1000 dilution for Western blot),

Secondary antibodies: FITC-labelled rabbit anti-mouse IgG (Dako, lot# 20035868, 1:200), HRP-conjugated rabbit anti-mouse IgG (Dako, F0261, lot# 20034868, 1:5000), HRP-conjugated Goat Anti-Rabbit IgG (Dako, F0448, lot# 20042622, 1:3000 dilution for Western blot), HRP labeled polymer conjugated Goat Anti-Rabbit IgG, (Dako, K4003, 1:300 dilution for IHC).

Validation

Commercially purchased antibodies against standard protein tags were tested on cell lines transfected with proteins with or without the relevant tag.

Eukaryotic cell lines

Policy information about cell lines

Cell line source(s)	CHOZN GS-/- (Sigma-Aldrich) and CHO-K1 (ATCC) cells were used for this study.
Authentication	No specific authentication of cell lines used apart from separate handling of original obtained vials throughout entire project. Each individual engineered CHO clones were however confirmed multiple times by CHO gene specific IDAA and Sanger sequencing in the target gene area(s).
Mycoplasma contamination	A representative set of growing cell lines in the lab selected randomly is subjected to mycoplasma screening bi-monthly, and within the last 10 yrs no infected cells have been found.
Commonly misidentified lines (See ICLAC register)	None of the cell lines used are listed in the ICLAC database.

Animals and other organisms

Policy information about studies involving animals; ARRIVE guidelines recommended for reporting animal research

Laboratory animals	Both knockout Fabry mice and WT controls used in this study have mixed genetic background of C57BL/6J and 129 strains with ~75 % of C57BL/6J strain background. Two-six months old male and female mice were used in study as specified in Methods and Figure Legends.
Wild animals	This study didn't involve any wild animals.
Field-collected samples	This study didn't involve samples collected from the field.
Ethics oversight	All animal procedures were reviewed and approved by the Institutional Animal Care and Use Committee of Baylor Research Institute.

Note that full information on the approval of the study protocol must also be provided in the manuscript.
